# Sources and traits of bacteria and fungi found in the near-surface atmosphere

**DOI:** 10.1128/aem.02348-25

**Published:** 2026-07-02

**Authors:** Claire C. Winfrey, Andrea A. Socualaya-Torres, Julian Resasco, Noah Fierer

**Affiliations:** 1 Department of Ecology and Evolutionary Biology, University of Colorado 118570, Boulder, Colorado, USA; 2 Cooperative Institute for Research in Environmental Sciences, University of Colorado 1877, Boulder, Colorado, USA; University of Minnesota Twin Cities, St. Paul, Minnesota, USA

**Keywords:** aeromicrobiome, air microbiome, bioaerosols, air fungi, air bacteria, phyllosphere, soil microbiome, trait-based ecology

## Abstract

Fungi and bacteria are ubiquitous in the near-surface atmosphere, and these airborne microbes can have important impacts on ecosystem and human health. Previous work has established that plant leaves and soils are the most likely sources of airborne microbes in natural, inland systems. Yet, the relative importance of these sources on the amounts and types of airborne microbes remain poorly quantified. Furthermore, not all microbes found in a source environment are equivalent in their ability to be aerosolized and persist in the near-surface atmosphere, but the specific traits associated with the capacity for aerial transport remain uncertain. To address these knowledge gaps, we collected 110 bioaerosol samples from the near-surface atmosphere in ~1 ha open savanna-like habitat fragments, and surrounding plantation forest in South Carolina, USA. We also collected samples from local leaves and soils to quantify potential bioaerosol sources. Despite the pronounced vegetation differences, the concentrations and composition of the airborne microbial assemblages in the distinct vegetation types did not differ, most likely due to substantial air mixing at this spatial scale. Foliar surfaces were more important sources of fungi and bacteria to the near-surface atmosphere than soils over the course of this study. Compared with foliar surfaces, bioaerosols were enriched in spore-forming bacteria and fungal taxa that produce fruiting bodies and smaller spores. Our work highlights that bioaerosols in the near-surface atmosphere are relatively unaffected by land-use change at small scales, and we can identify microbial traits that are important determinants of atmospheric dispersal.

**IMPORTANCE:**

Each year, an estimated 10^23^ fungal spores and 10^24^ bacterial cells enter the atmosphere from terrestrial sources. These airborne microorganisms have important effects on human health and ecosystem processes, with atmospheric transport serving as a key mode of dispersal that shapes microbial distributions. We paired analyses of microorganisms in bioaerosols and local sources to address important outstanding questions about the spatial variation, sources, and traits of airborne microorganisms. We establish that the largest local sources of airborne microbes in our system are leaf surfaces, yet not all leaf-associated bacterial and fungal taxa are equally capable of dispersal through the atmosphere. Furthermore, we identified specific bacterial and fungal traits that facilitate microbial aerosolization and persistence in the atmosphere, building toward a more mechanistic understanding of microbial aerial dispersal.

## INTRODUCTION

The atmosphere is not an easy place to be a microbe. Stressors include desiccation, DNA-damaging ultraviolet radiation (UV), rapid temperature fluctuations, and limited resources to sustain microbial activities (1–3). In spite of these challenging conditions, bacteria, archaea, and fungi are found in the atmosphere, with an estimated 10^23^ fungal spores (4) and 10^24^ bacterial cells (5) emitted from Earth's surface to the atmosphere annually. These airborne microorganisms, i.e., the “aeromicrobiome,” link metacommunities of microbes across terrestrial source environments, at the same time playing integral roles in human and environmental health (1–3). For example, viable plant and animal pathogens are routinely cultured from outdoor dust samples (6), and there is increasing evidence that important plant fungal partners, including some mycorrhizal taxa, are adapted for dispersal through the air (7). Because aerial transport likely explains why many microbial taxa are found across such large spatial extents (1, 3, 8), elucidating the patterns and determinants of the aeromicrobiome is important for understanding the biogeography of microorganisms across different ecosystems and scales.

Despite the central role the atmosphere plays in microbial dispersal, we lack a cohesive understanding of the contribution of local sources to the aeromicrobiome. Previous work has shown that specific microbes found in the near-surface atmosphere often overlap with those found on the foliar surface of plants and in the soil (9–12). However, we know of no studies that have specifically tried to quantify the relative contributions of soil vs foliar surfaces to the aeromicrobiome, as doing so requires sampling of the microbes in these potential source environments and comparing those microbial assemblages to those found in co-located bioaerosol samples. The relative importance of these two terrestrial sources (soil and foliar surfaces) is expected to differ depending on the ecosystem in question (10). For instance, dust is a major vector of soil bacteria from desert systems where wind erosion of surface soils can be substantial (13, 14), but in landscapes with more vegetation and less exposed soil, we would expect a greater contribution of leaf-derived microbes to the near-surface atmosphere. Furthermore, airborne bacteria and fungi may have different origins even in the same ecosystem, given differences in size, dispersal mechanisms, and distributions. For example, Lymperopoulou et al. (12) found that leaf-associated bacteria are more likely to become airborne than leaf-associated fungi, but we also know that there are certain fungal plant pathogens that can disperse far from leaf surfaces through the atmosphere (6, 15). In short, it is challenging to predict *a priori* which specific source environments are most likely to contribute to the aeromicrobiome and how the relative importance of these sources might differ across taxa.

The composition of the aeromicrobiome often varies depending on the surrounding ecosystem or land cover type (10, 16–19). However, most studies have compared aeromicrobiomes across large spatial scales (typically tens to hundreds of kilometers), so we do not know the scale at which these differences become apparent. It is possible that the aeromicrobiome shifts at sub-kilometer scales in response to changes in land cover, as can occur in fragmented landscapes where distinct soil edaphic characteristics (e.g., 20) and vegetation types (e.g., 21) can occur in close proximity. Since the soil microbial communities in habitat fragments often differ from those found in the surrounding matrix (22–24) and plants often harbor species-specific foliar surface communities (25, 26), the aeromicrobiome might differ inside versus outside habitat fragments, even if those fragments are relatively small. Alternatively, even if the foliar and soil microbial communities differ in the distinct habitat types, there may be sufficient air mixing, especially in high wind or turbulent conditions, to homogenize the aeromicrobiome and obscure any potential source-specific impacts on the aeromicrobiome at smaller spatial scales (27). If so, the aeromicrobiome of a particular land use type would likely be different in a fragmentation context than it would be otherwise. Understanding the scale at which the aeromicrobiome changes across habitat boundaries is particularly important for understanding microbial dispersal and spillover (sensu [28]) across fragmented landscapes (24) which are increasingly common across the globe due to human activities (29).

From any given source environment, not all fungi and bacteria are equally likely to be found in the air. Comparing bioaerosol samples to samples taken from probable local sources makes it feasible to identify traits associated with the likelihood that a given taxon will become aerosolized and persist in the atmosphere. There are numerous traits that may be associated with airborne dispersal, including the presence of oxidative stress and UV repair enzymes (30), fungal spore ornamentation (31), or biofilm formation (30, 32). However, we focus here on four traits that we expect to be particularly important and which are reasonably well-represented in fungal and bacterial trait databases: the capacity for fungi to produce aboveground fruiting bodies, fungal spore size, bacterial sporulation, and bacterial pigmentation. First, aboveground fungal fruiting bodies facilitate aerosolization and serve as a surface from which spores can be passively carried by the wind (33, 34), meaning that fungi that produce aboveground fruiting bodies may be particularly adapted for the release stage of aerial dispersal. Second, fungi that produce smaller spores may have a greater potential for atmospheric transport than fungi that produce larger spores because, all else equal, smaller spores should remain aloft in the atmosphere longer and travel farther (7, 35). Third, bacterial sporulation is likely to increase survival in the harsh atmospheric environment by conferring tolerance to UV radiation, desiccation, and other stressors (2, 30). Finally, bacterial pigmentation should enhance survival in the near-surface atmosphere by protecting cells from UV radiation and minimizing oxidative damage (36–38).

Our study addressed three main questions. First, what is the dominant source of bacteria and fungi to the near-surface atmosphere (soil vs. foliar surfaces)? Second, are differences in vegetation types across fragmented landscapes associated with local-scale variation in the aeromicrobiome? Third, which bacterial and fungal traits differentiate the taxa detected in the near-surface atmosphere from those in the dominant source environments? To address these questions, we surveyed the aeromicrobiome and potential plant and soil sources in an experimentally fragmented landscape in the southeastern USA, collecting bioaerosol samples from paired locations in open savanna-like patches (13,750 m^2^ in size) and the surrounding forested matrix. We used marker gene sequencing coupled with quantitative PCR to characterize the types and total amounts of bacteria and fungi in the bioaerosol samples, directly comparing the specific taxa found in the bioaerosol samples to those found in corresponding foliar surface and soil samples collected from the same area. Finally, we leveraged microbial trait databases to infer and then compare fungal spore size, fungal fruiting body morphology, bacterial sporulation, and bacterial pigmentation between taxa enriched in the aeromicrobiome and the corresponding source environment.

## MATERIALS AND METHODS

### Sample collection

**Fig 1:**
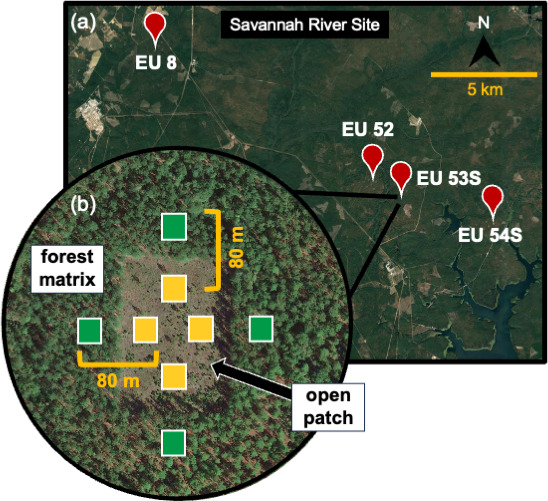
Satellite images showing the experimental landscape at the Savanah River Site, South Carolina, USA (**a**), and an aerial view of one of the four experimental unit (EU) study sites (**b**). In panel a, red placemarks indicate each of the four replicate EUs on the landscape. In panel b, yellow and green boxes represent the four locations in the open patch and forested matrix, respectively, of the eight UPAS air samplers that were deployed each 24-h sampling period across EUs. Satellite images were sourced from Google Earth (image ©2026 Vexcel Imaging US, Inc., and image ©2026 Airbus).

This work was conducted at the Savannah River Site (SRS) located in southwestern South Carolina, USA (33°16′ N, 81°37′ W; Fig. 1). The SRS is a 1,240 km^2^ U.S. Department of Energy (DOE) National Environmental Research Park that is managed by the U.S. Forest Service (Fig. 1). We worked at four replicate 100 × 137 m open-habitat patches (hereafter “experimental units,” EUs) that were formed by clearing mature pine plantation in 1999, 2000, and 2007 as a part of the SRS Corridor Project (see reference 39 for additional details). These open patches feature grasses, forbs, and a low density of longleaf pines, and efforts are underway to restore the patches to longleaf pine savanna (40), the historical ecosystem in this region (41). Surrounding each patch is a forested matrix dominated by mature loblolly pine (*Pinus taeda*) and longleaf pine (*P. palustris*) plantation with some mixed hardwoods and shrubby undergrowth (39, 40).

In June and July 2022, we collected bioaerosol samples from each of our four replicate EUs on 4 separate days, for a total of 16 sampling days. We worked to minimize temporal autocorrelation in our bioaerosol samples by randomly selecting the dates on which each EU was sampled (Table S1). On each sampling date, eight bioaerosol samples were collected from paired locations 80 m apart in the forested matrix and open patch using UPAS active air samplers (v2, Access Sensor Technologies [AST], Fort Collins, CO, USA) fitted with AST open inlets and cartridges (i.e., without particle size selection) and 3 µm Teflon filter membranes (Measurement Technology Laboratories, Minneapolis, MN, USA) (Fig. 1). The UPAS samplers were positioned face down (to minimize dust deposition) at a height of 2 m and sampled 2 L min^−1^ for 24 h. We cleaned the UPAS samplers between deployments with a 3% bleach solution and ethanol. To check for potential contamination, we prepared a “bioaerosol field blank” on each sampling day by placing a new Teflon filter into a freshly cleaned UPAS that was run for 5 min.

We deployed a WindLog Wind Data Logger (RainWise Inc., Boothwyn, PA, USA) at a height of 0.91 m in the center of each open patch to collect average and maximum gust wind data at 15-min intervals for each 24-h sampling period. We obtained air temperature, humidity, and rainfall data at 15-min intervals from the Savannah River National Lab (SRNL) Atmospheric Technologies Group (ATG). The SRNL ATG weather stations are located near the center of the SRS, ranging from about 9.5 to 14.5 km from our sites.

In addition to the bioaerosol samples, we collected foliar surface and soil samples from our study sites. To collect foliar surface samples, we filled 24 oz. sterile bags (Whirl-Pak, Pleasant Prairie, WI, USA) with undamaged leaves or blades from the top 12–15 species by abundance in each EU (unpublished data from Lars Brudvig and Ellen Damschen), for a total of 59 samples across 30 species of trees, grasses, and herbaceous plants (see Table S2 for a list of each plant species collected per EU). Leaves and blades were collected from mature plants in a haphazard manner throughout the EU, including in the patch and matrix if present. We used a fresh pair of nitrile gloves for each plant sample and collected all foliar surface samples in the same week of sampling in July 2022. We adapted our leaf washing protocol from Redford et al. (25). On the same day as collecting plant material in the field, we added 50 mL of a wash solution (1:50 dilution of 1 M Tris-HCl, 500 mM EDTA, and 1.2% Triton diluted in sterile, Cytiva HyPure cell culture grade water adjusted to pH 8.0 and 0.2 µm sterile-filtered) to each foliar surface sample. After shaking the plant material in the wash solution for 60 s, we transferred 10 mL of the wash solution into a 15 mL sterile conical tube which was frozen at −20 °C until DNA extraction. To test for potential contamination introduced by this washing process, we prepared “foliar surface field blanks” by repeating the washing procedure above in fresh Whirl-Pak bags without leaves (*n* = 3). We collected 40 soil samples from each EU in May and June 2021 as a part of a previous study (24). Since soil communities unexposed to extreme disturbance events tend to be stable from year to year (e.g., 42, 43), using soil samples collected 1 year prior would be unlikely to affect our results. Briefly, the soil sampling design featured four 100-m transects extending from the center of the open patch to 50 m into the forested matrix. Soil samples were collected at 10-m increments along the transects, with each sample consisting of 8 subsamples of mineral soil collected from 0 to 5 cm which were sieved to 2.0 mm, homogenized, and frozen at −20°C until DNA extraction.

### DNA extraction

We used a DNeasy PowerSoil Pro Kit (Qiagen, Germantown, MD, USA) to extract DNA from bioaerosol and foliar surface microbiome samples (the same method used for extraction of DNA from the soil samples, see [24]). We performed DNA extractions for bioaerosol and foliar surface samples in different batches to minimize potential cross-contamination. We extracted DNA from bioaerosols as in Gering et al. (44), performing all DNA extraction steps while wearing personal protective equipment (PPE) in a cleaned laminar flow hood, including one half of the filter in each extraction, and eluting DNA in 50 µL at the final step in the protocol. In each batch of bioaerosol extractions, we also extracted DNA from the 0.2 µm sterile-filtered phosphate-buffered saline and 1% Tween-80 solution used to pre-treat filters (see reference 44 for more details), i.e., a “wash buffer blank.” To extract foliar surface DNA, we again used a DNeasy PowerSoil Pro Kit, adding 800 µL of each collected foliar surface wash solution to a PowerBead tube. We then followed the manufacturer’s standard protocol, except we added 50 µL of the elution buffer to the spin column in the penultimate step and added a 5-min room temperature incubation before centrifugation. To check for potential contamination introduced during extraction, we included a DNA extraction blank in each batch of bioaerosol and foliar surface DNA extractions.

### Marker gene amplicon sequencing

To determine the bacterial diversity in our samples, we amplified the V4 region of the 16S rRNA gene with universal primers 515-F and 806-R (45). We used primers ITS1-F and ITS2-R to amplify the internal transcribed spacer region (ITS) of fungi in our samples (46). Each 25 µL reaction consisted of 12.5 µL Platinum II Hot-Start Master Mix (Invitrogen, Waltham, MA, USA), 10.5 µL PCR grade water, 0.5 µL of each 10 µM primer, and 1 µL template DNA. For the bioaerosol 16S rRNA gene PCRs only, we increased the template DNA to 2 µL and decreased the PCR grade water to 9.5 µL. PCR cycling conditions were the same as in Winfrey et al. (24). PCRs were performed in duplicate, and amplicon lengths were visualized with gel electrophoresis. Amplicon libraries were generated separately for the air and foliar surface samples to avoid potential cross-contamination. We used SequalPrep Normalization Plate Kits (Applied Biosystems, Waltham, MA, USA) to clean and normalize all samples and then pooled samples in equimolar concentrations. We sequenced amplicon libraries (i.e., foliar surface 16S, foliar surface ITS, bioaerosol 16S, and bioaerosol ITS) on four separate Illumina MiSeq runs at the Center for Microbial Exploration at the University of Colorado Boulder, using 2 × 150 bp cycle kits for 16S rRNA sequencing and 2 × 250 bp cycle kits for ITS rRNA sequencing. In total, we sequenced 233 samples and controls, consisting of 126 bioaerosol samples, 16 bioaerosol field blanks, 11 bioaerosol wash buffer blanks, 59 foliar surface samples, 3 foliar surface field blanks, 14 DNA extraction blanks (10 and 4 associated with bioaerosol and foliar surface extractions, respectively), and 4 no-template controls (2 associated with both bioaerosol and foliar surface PCRs). The 160 soil samples included from Winfrey et al. (24) were prepared using primer sets and PCR cycling conditions identical to those described above.

### Quantitative PCR

To estimate bacterial and fungal DNA concentrations in the collected bioaerosol samples, we performed quantitative PCR (qPCR). We replicated the methods of Gering et al. (44), except that we included 2 µL of template DNA for each reaction. In brief, we used the primers 515f/806r to target the bacterial/archaeal 16S rRNA gene and primers FF390f/FR1r to target the fungal ITS region. To generate standard curves, we used purified genomic DNA from *Escherichia coli* DH10B and *Aspergillus fumigatus* (ATCC MYA-4609D-2). We calculated the 16S rRNA gene copies and ITS region copies in each bioaerosol sample based on the standard curves, assuming 7 16S rRNA copies per genome of *E. coli* (47) and 38 ITS region copies per genome for *A. fumigatus* (48). Results are reported for each bioaerosol sample as the number of 16S rRNA or ITS copies per m^3^ of air sampled.

### Bioinformatics

All analyses were conducted in R (v4.5.1 [49]), and unless otherwise noted, the 16S rRNA and ITS rRNA data sets were processed separately using the same pipeline. Bioinformatics were performed as in Winfrey et al. (24), except for a few modifications described below. In brief, we first demultiplexed our reads and removed primers, using the programs idemp (https://github.com/yhwu/idemp) and cutadapt (v1.8.1 [50]). For quality filtering, denoising, dereplicating, and merging of raw paired-end MiSeq reads, we implemented DADA2 (v1.22.0 [51]). Bioaerosol, foliar surface, and soil sample sequence data were processed separately through the denoising step of the DADA2 pipeline; after denoising, all sequence data were processed together to generate a single table of ASVs (amplicon sequence variants, clustered at 100% similarity). ASVs were classified using the “assignTaxonomy” function in DADA2 against the SILVA reference database (v138.1 [52–54]) for 16S rRNA gene reads and against the UNITE reference database for ITS region reads (v8.3 [55–57]).

We implemented several steps to remove non-focal or potential contaminant ASVs in our data sets. First, we note that we excluded bioaerosol samples that failed due to UPAS malfunctioning in the field (*n* = 16), resulting in the initial retention of 110 samples with a minimum runtime of 23.4 h. For the 16S rRNA data set, we removed chloroplasts, mitochondria, and any ASVs not assigned to at least the phylum level. In addition, we removed archaea, as archaea were rare (< 0.1% of 16S rRNA gene reads in bioaerosol samples) and were not the focus of this study. For the ITS data set, we removed all non-fungal reads as well as any ASVs not taxonomically classified to at least the phylum. We identified probable lab-based contaminants as any ASV that occurred in at least half of bioaerosol-associated blanks (i.e., 19/39 blanks) or foliar surface blanks (i.e., 4 out of 7 or 9 blanks; foliar surface no-template PCR controls had no 16S data following the DADA2 pipeline due to too few reads) and removed these ASVs from associated samples for all downstream analyses. Bioaerosol-associated contaminants included 10 contaminant bacterial ASVs, comprising a median of 21.2% of reads across all blanks, compared to a median of 2.5% across all 110 bioaerosol samples (Table S3). Foliar surface-associated contaminants included five bacterial ASVs, accounting for a median 6.4% of reads in blanks and 1.9% of reads in samples before removal (Table S3). No fungal ASVs were identified as contaminants. At this stage, the median 16S rRNA read counts across samples or blanks were 10,583 per bioaerosol sample, 2,077 per bioaerosol control, 23,587 per foliar surface sample, and 1,083 per foliar surface control. For our ITS data set, median read counts across sample or blanks were 35,188 per bioaerosol sample, 582 per bioaerosol control, 42,182 per foliar surface sample, and 290 per foliar surface control. We next removed any samples or blanks that had fewer than 5,500 16S reads or 8,500 ITS reads and then rarefied the remaining samples to these thresholds. Unless otherwise noted, we used these rarefied data for all analyses. We rarefied because read depth varied by one to two orders of magnitude (within and among sample types), and rarefying has been shown to produce accurate dissimilarities among samples (58) and reduce the false discovery rate in differential abundance inference when read depths are similarly uneven (59). For bioaerosol samples only, we removed singleton and doubleton ASVs. After these quality filtering steps, we retained 84 bacterial bioaerosol samples, 58 bacterial foliar surface samples, 157 bacterial soil samples, 110 fungal bioaerosol samples, 59 fungal foliar surface samples, and 155 fungal soil samples. We note that these quality filtering steps left 11 and 2 out of 48 total blanks still remaining in our 16S and ITS data sets, respectively, which were virtually all field-associated controls (see Table S3 for additional details).

### Determination of microbial sources

To compare the importance of the two source environments (foliar surfaces versus soil) to the air microbiome, we first performed indicator species analyses to determine ASVs that were associated with foliar surface or soil samples. Specifically, we used the “multipatt” function from the “indicspecies” R package (v1.8.0 [60]), specifying func = “r.g.,” implementing 9,999 permutations, and applying a *P* value threshold of 0.05. Next, we determined the likely source of the aeromicrobiome by comparing soil and foliar surface indicator taxa (as proportion of reads and as proportion of distinct ASVs) in our bioaerosol samples with Wilcoxon signed-rank tests.

### Differences in the air microbiome based on habitat

We investigated our hypothesis that the air microbiome would be different in the forested matrix and the open patch by analyzing differences in community composition and testing for differences in microbial DNA concentrations between habitats. To test if community composition differed among the forested matrix and the open patch, we performed PERMANOVAs (61, 62) on Bray-Curtis dissimilarities with the “adonis2” function with 9,999 permutations (vegan R package, v2.7.1 [63]). To account for the influence of EU in these tests, we used sequential sums of squares so that variation due to EU was accounted for before considering habitat type and we restricted permutations (“permute” function, v0.9.8 [64]), to occur only within each EU, as suggested in Bakker (65). To restrict our permutations, it was necessary to have each combination of EU and habitat type have the same number of samples, so we randomly selected 12 and 7 samples from each EU/habitat type combination from our fungal and bacterial data sets, respectively. To assess the effects of habitat type on bacterial and fungal DNA concentrations in the bioaerosol samples, we implemented generalized linear mixed models (GLMMs) with a negative binomial distribution (“glmmTMB” function from glmmTMB package, v1.1.12 [66, 67]), testing model significance with Wald Chi-Square tests (“Anova” function from the car package, v3.1.3 [68]). For both fungal and prokaryotic GLMMs, we included nested random effects of sampling day within EU.

### Trait-based analyses of air-specializing taxa

To test our hypotheses that the near-surface atmosphere would disproportionately harbor fungi and bacteria with particular traits compared to the source environments (in this case, foliar surfaces, see Results and Discussion), we first identified taxa that were differentially abundant in the near-surface atmosphere and on plant leaves by performing an analysis of composition of microbiomes with bias correction 2 (ANCOM-BC2) on unrarefied bioaerosol and foliar surface samples. We used unrarefied data for this analysis because the ANCOM-BC2 algorithm models and corrects for uneven sampling depth (69). Specifically, we used the “ancombc2” function with sample type as the fixed effect, struc_zero = TRUE, and otherwise default parameters (ANCOMBC R package, v2.10.1 [69–71]). We considered differentially abundant taxa as those that passed ANCOM-BC2’s pseudo-count sensitivity test with an adjusted *P* value < 0.05. Furthermore, in its respective group (i.e., bioaerosol or foliar surface samples), a differentially abundant ASV had to occur in at least 10% of samples and comprise at least 0.05% of total reads.

We next compared how these bioaerosol-enriched and foliar surface-enriched ASVs differed in various traits, namely, spore volume and fruiting body morphology for fungi and the potential for bacteria to sporulate and be pigmented. We sourced data on fungal spore volume from reference 72 (accessed 29 May 2024 from https://github.com/aguilart/Symbiotic-status-and-fungal-spore-size), focusing only on uninucleate sexual spores (i.e., meiospores, including ascospores and basidiospores), as there were limited data available for multinucleate or asexual spores. To do so, we matched our ASVs identified using ANCOM-BC2 to those in the database at the species taxonomic level, or genus level if a match could not be made at the species level, and then we took the median spore volumes of these matches to infer ASV spore volumes (hereafter referred to as the “full spore volume” data set). Because these database matches for a given ASV sometimes varied widely in spore volume, we also created a subset of the matches that included only those ASVs for which the matches in spore volume closely agreed (max volume − min volume)/median volume <0.15, henceforth referred to as the “subsetted spore volume data set”). Next, to test if bioaerosol-associated and foliar surface-associated ASVs differed in their spore volumes, we used Wilcoxon rank-sum tests, performing separate tests on the full and subsetted spore volume data sets. For fungal fruiting body morphology, we matched our ASVs to those in FUNGuild (v1.1 [73]), considering only “probable” or “highly probable” ecological guild classifications. We inferred the ability of bacterial ASVs to form spores by utilizing the vsearch tool (v2.15.2 [74]) to match bioaerosol- and foliar surface-associated ASVs identified with ANCOM-BC2 to taxa in the Madin et al. (75) database, considering sequences that were at least 97% identical over the aligned V4 16S region as matches. To infer pigmentation in bacterial ASVs, we matched our ANCOM-identified ASVs to those in Barberán et al. (76) at the species taxonomic level, or genus level if a match could not be made at the species level. We categorized an ASV as “spore-forming,” “non-spore-forming,” “pigment-producing,” or “non-pigment-producing” only if all matches in the respective trait database were classified as such. We categorized an ASV as a “possible” spore-former or pigment-producer if it matched to both taxa with and without these traits in the respective databases. Using this inferred information on sporulation and pigment producing abilities, we performed Fisher exact tests to test our predictions that bioaerosol samples would feature a greater proportion of “possible” or “spore-forming” bacteria and “possible” or “pigment-producing” bacteria than foliar surface samples.

## RESULTS AND DISCUSSION

### Overview of the aeromicrobiome and environmental conditions

Across the collected bioaerosol samples that met our criteria for inclusion in downstream analyses (*n* = 84 bacterial samples, *n* = 110 fungal samples), estimated DNA amounts ranged from 10^3^–10^5^ 16S rRNA copies per m^3^ for bacteria and 10^4^–10^7^ ITS region copies per m^3^ air for fungi. These concentrations are similar to airborne bacterial and fungal concentrations observed in other studies of the aeromicrobiome (9, 11, 12, 77, 78). However, in contrast to other studies that used similar molecular methods (12, 77), we found that the airborne fungal concentrations exceeded bacterial concentrations in nearly all cases (Table S1). These differences could be attributed to differences in bioaerosol sampling methodologies or, more likely, are simply a product of the sampling time and location given that our study site was highly vegetated and samples were collected during a very humid and warm period (Fig. S1 and Table S1).

**Fig 2:**
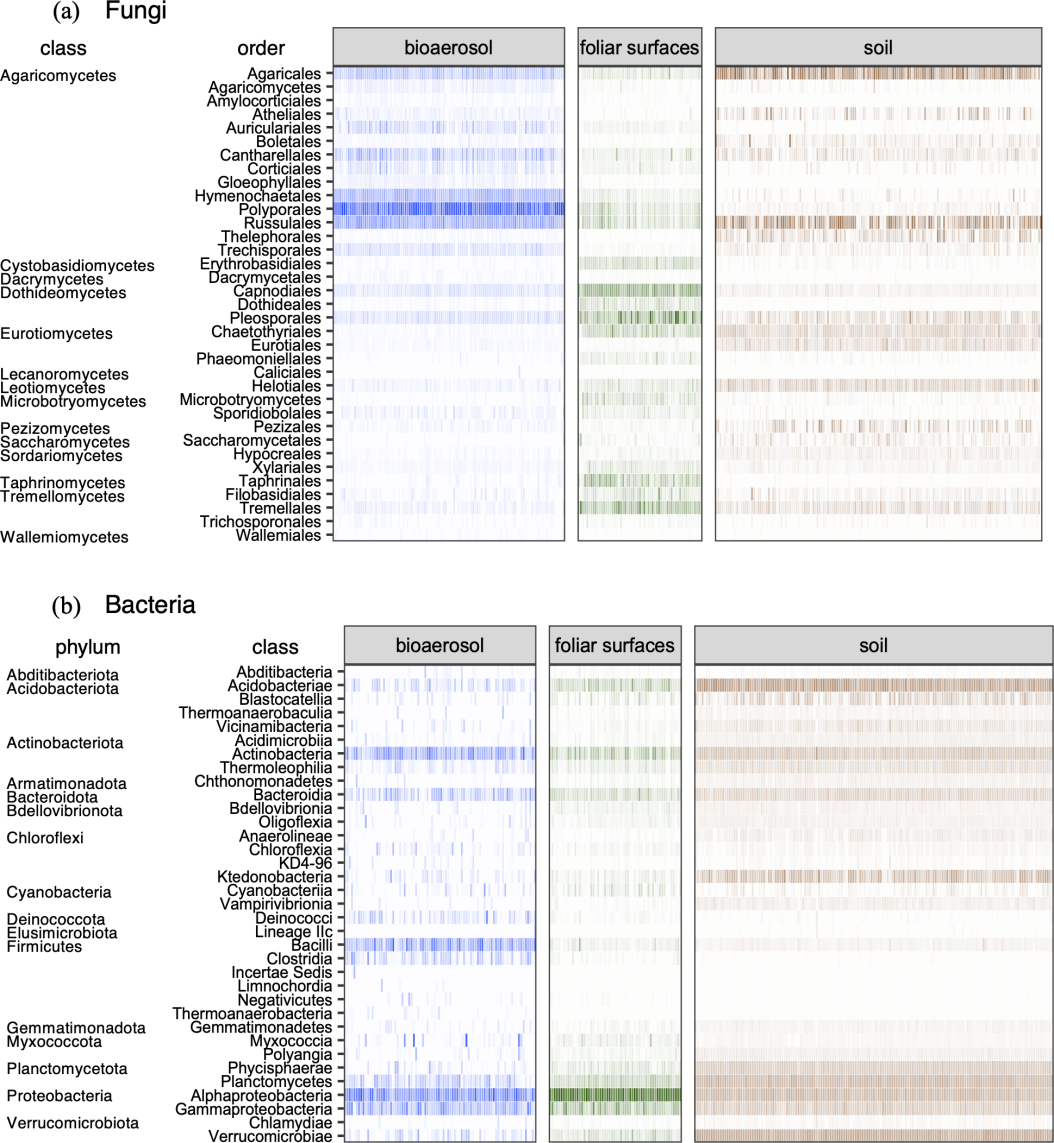
Heat maps showing the relative abundances of the 35 fungal orders (**a**) and bacterial classes (**b**) that were most abundant in bioaerosol samples, across bioaerosol, foliar surface, and soil samples. Columns correspond to individual samples. In our fungal and bacterial data sets, respectively, there were 110 and 84 bioaerosol samples, 59 and 58 foliar surface samples, and 155 and 157 soil samples. The darker the color, the more abundant the taxonomic group in the sample. Relative abundance values have been square-root transformed for greater visibility on the plot.

We found minimal variation in the general composition of the bacterial and fungal bioaerosol assemblages across our sampling campaign, which spanned 3 weeks and included four different sites separated by ~1.2–18.5 km. The top fungal orders and their mean relative abundances in bioaerosol samples included Polyporales (49%), Russulales (14%), and Hymenochaetales (11%) (Fig. 2a, Table S4). The bacterial assemblages were dominated by classes Alphaproteobacteria (27%), Bacilli (15%), and Actinobacteria (14%), as shown in Fig. 2b and Table S5. These groups of bacteria and fungi have also been observed to be dominant in other studies of the aeromicrobiome (9, 16). Although the goal of this study was not to quantify variation across the sites or temporal variation, we note that the same set of fungal and bacterial taxa were found to be dominant in all of the bioaerosol samples collected from the four sites (EUs) over the 3-week period (Fig. 2). This relative consistency in the overall structure of the bacterial and fungal assemblages is to be expected given the landscape-level homogeneity and the relatively close proximity of the four sites. In addition, weather conditions were reasonably consistent across the duration of the sampling effort, characterized by calm wind speeds of 0.5–2 km per hour, mean daily temperatures of 25–30°C, and mean daily humidities typically above 65% (Fig. S1 and Table S1). However, at finer taxonomic levels, there was some apparent variation in the composition of the aeromicrobiome, so these microbial assemblages in the near-surface atmosphere are not homogeneous (Fig. 2; Fig. S2). While we discuss specific ASVs of interest in later sections, it is important to note that the most abundant bacterial ASV in our bioaerosol samples belonged to *Staphylococcus* (present in 72.6% of non-control bioaerosol samples, with a mean of 2.1% of 16S rRNA gene reads, Table S5). While *Staphylococcus* is often found on the skin of humans and other animals (79), *Staphylococcus* is unlikely to represent a contaminant in our study. Most importantly, out of the 39 bioaerosol-associated blanks (field blanks, wash buffer blanks, DNA extraction blanks, no-template controls), this ASV was not detected in 26 of these controls and was very rare in the other remaining blanks (<11 reads per blank). Moreover, there is mounting evidence that members of *Staphylococcus* are endemic to a wide range of environments including plant sources (80 and references within).

### Leaf surfaces, not soil, are the dominant contributor to the air microbiome

**Fig 3:**
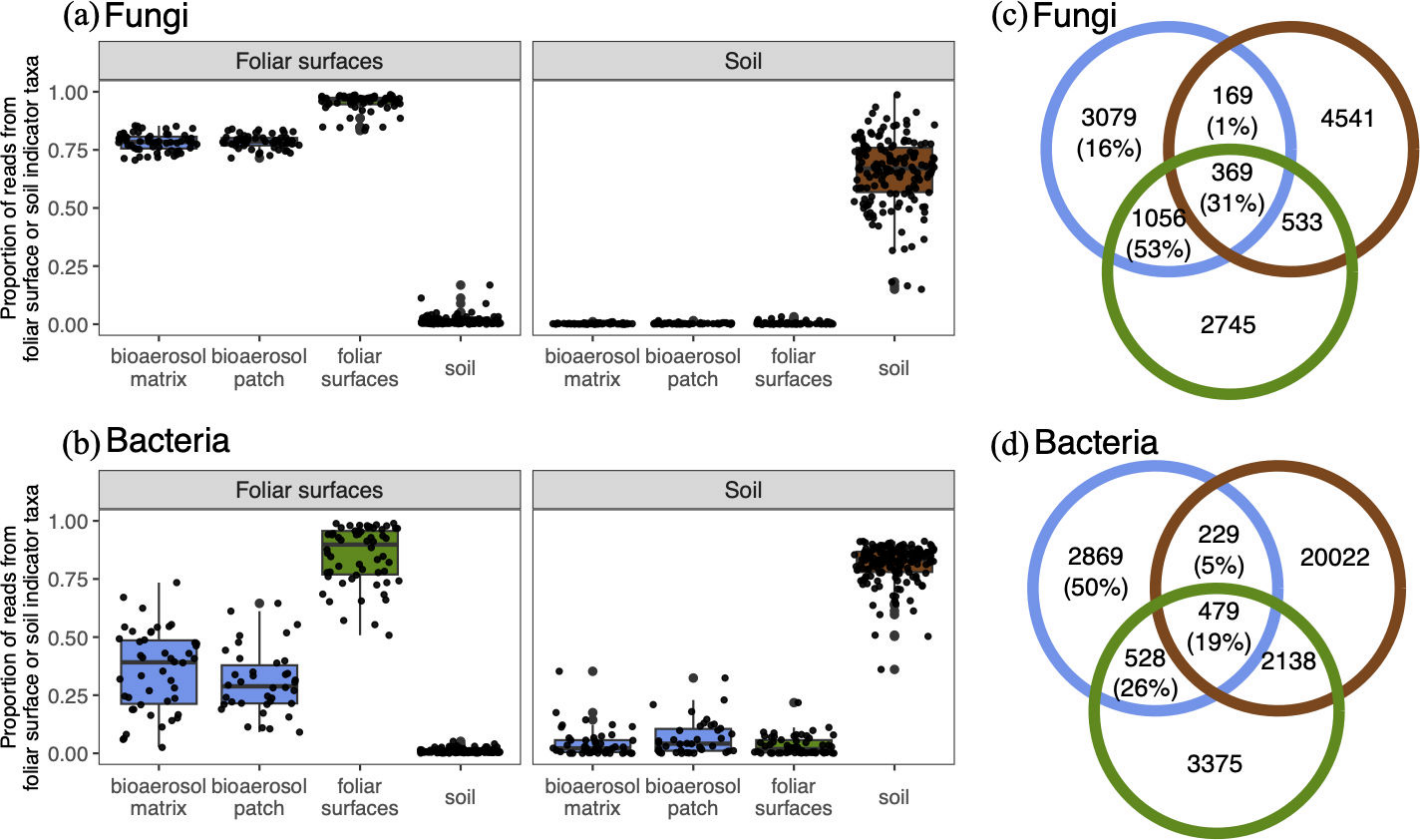
Associations among taxa in bioaerosols and the potential source environments of foliar surfaces and soil. Panels **a** and **b**, respectively, show the proportion of reads from foliar surface or soil indicator taxa in each fungal and bacterial bioaerosol sample. Bioaerosol samples featured a larger proportion of reads from foliar surface than soil indicator taxa (Wilcoxon signed-rank tests: *V* = 6,105 for fungi and *V* = 3,484 for bacteria, *P* < 0.001 for both). However, the proportion of soil-associated taxa (as reads) was higher in the open patch than in the forested matrix (two-proportions *z*-test: fungi: *χ*^2^ [1, *N* = 734,381 reads] = 25.3, *P* < 0.001; bacteria: *χ*^2^ [1, *N* = 178,956 reads] = 1,665.3, *P* < 0.001). The Venn diagrams indicate the number of ASVs unique to and shared among bioaerosol (blue), soil (brown), and foliar surface (green) samples in fungal (**c**) and bacterial (**d**) samples. Values in parentheses indicate the percentages of total bioaerosol reads that are represented by each subset of ASVs.

As expected, the bioaerosol, foliar surface, and soil samples had distinct bacterial and fungal assemblages (Fig. S2). However, as hypothesized, foliar surfaces, not soil, were the dominant source of microbes in the near-surface atmosphere in our system (Fig. 3). Both fungi and bacteria in the bioaerosol samples showed a much stronger signal of foliar surface-associated than soil-associated taxa, with both a higher proportion of reads (Fig. 3a and b) and individual ASVs (Fig. S3) in bioaerosols contributed by foliar surface associated taxa. The fungal reads in bioaerosol samples consisted of a median of 78.5% foliar surface-associated and 0.2% soil-associated taxa (Fig. 3a), whereas bacterial reads per bioaerosol sample had medians of 31.7% foliar surface-associated and 2.9% soil-associated taxa (Fig. 3b).

There are two reasons why the foliar surface- and soil-associated taxa together do not add up to 100% of bioaerosol reads. First, many taxa were detected in similar abundances in the foliar surface and soil samples, meaning that we could not distinguish between soils and plants as the likely origin of these taxa. This may partially explain why we observed a stronger influence of foliar surface-associated taxa in fungal bioaerosol reads than in bacterial bioaerosol reads (see above). As a greater percentage of the ASVs found on foliar surfaces were also detected in the soil among bacterial samples (39.9%, Fig. 3d) than among fungal samples (19.2%, Fig. 3c), we were better able to distinguish foliar surface-associated taxa from soil-associated taxa in our fungal indicator species analysis than in our bacterial analysis. Second, there were some taxa present in the bioaerosols that were not found in either the sampled soils or foliar surfaces, particularly for bacteria (65.9% of fungal and 69.9% of all bacterial ASVs detected in the bioaerosol samples, with these ASVs representing 15.6% and 50.3% of total ITS and 16S rRNA reads across bioaerosol samples, respectively; Fig. 3c through d). These taxa may have originated from local or distant plant species or soils that we did not sample, or from other known emission sources, such as distant aquatic (2, 81) or anthropogenic (82–84) environments. Alternatively, it is possible that these taxa were present in sampled soils or foliar surfaces, but their abundances were too low to be detected.

Given the densely vegetated nature of the sampling sites (see vegetation measurements in 24) and our summer sampling time, our finding that foliar surfaces contributed more bacteria and fungi to the aeromicrobiome than soil is not surprising. Accordingly, taxa previously reported to be common on foliar surfaces and which we identified as foliar surface indicator taxa comprised a large proportion of the reads within bioaerosol samples. A notable bacterial example is the leaf-specializing genus *Methylobacterium-Methylorubrum* (85–87), represented in our bioaerosol samples by two ASVs detected in 74% and 57% of the bioaerosol samples (; Table S5). A fungal example is the genus *Sporobolomyces*, which is a dominant colonizer of the foliar surfaces of many species of plants (86, 88) and which was detected in 61% of all bioaerosol samples (Table S4). However, we do not claim that foliar surfaces necessarily contribute more airborne bacteria and fungi to the near-surface atmosphere than soils at other times of year or in other systems. First, because fungal and bacterial taxa in bioaerosols often vary seasonally (7, 89, 90), it is possible that we would observe a different degree of overlap between the microbes in bioaerosols and foliar surfaces or soils had we sampled a different time of year. Second, we would expect soil to be a more important source in areas where exposed ground is more common and vegetation is sparser, such as arid environments. In line with this prediction, our study provided some evidence that greater exposed soil would mean more soil-derived microbes in the air. Namely, despite our finding that foliar surfaces were the greater influence in both the patch and the matrix, the proportion of reads from soil-associated taxa was higher in the patch than in the matrix (Fig. 3). One explanation for these results is simply that the density of foliar surfaces is higher in the forested locations than in the open patch locations where small areas of bare ground were more evident (24).

### No differences in the air microbiomes across habitat types

We next hypothesized that there would be local-scale differences in the aeromicrobiome, with air samples collected from the forested matrix and the open patches having distinct airborne microbial assemblages. This hypothesis arises from the distinct plant communities (39, 40) and soil microbial communities (24) found in the open patches vs the surrounding forested matrix, as well as the observation that distinct plant taxa often harbor distinct bacterial and fungal communities on their foliar surfaces (25, 26). However, this hypothesis was not supported by our data. The total concentrations of bacterial and fungal DNA did not differ between bioaerosol samples collected from the forested matrix and open patch locations (Fig. S4 and Table S1). Likewise, the composition of the bacterial and fungal assemblages did not differ between the bioaerosol samples collected from the two habitat types (PERMANOVAs: *P* = 0.81 for fungi and *P* = 0.57 for bacteria [Fig. S4 and Table S6]). These results contrast with other studies which have documented habitat-specific differences in aeromicrobiome composition at larger spatial scales (10, 16–19). A likely explanation for our results is that there is simply sufficient mixing of air at the ~80 m scale we considered to mask any potential differences in the aeromicrobiome between the two habitats even though wind speeds were relatively low during the sampling period (Fig. S1 and Table S1). A next step would be to sample the aeromicrobiome at varying distances along transects extending further into the interior of each habitat type to determine the spatial scale at which differences in the amounts and types of microbes in the near-surface atmosphere may become apparent and how meteorological conditions impact potential mixing of bioaerosols across habitat types.

### The atmosphere selects for different taxa and traits than the leaf surface source

Given our finding that the foliar surfaces of plants were the primary source of fungi and bacteria found in the near-surface atmosphere, we next sought to determine which particular taxa were enriched in the atmosphere compared to this source environment and which traits may confer a selective advantage for dispersal and survival in the atmosphere. We did so by identifying taxa that were differentially abundant across the two environments (foliar surfaces vs the atmosphere, see Materials and Methods) and comparing inferred trait values for the differentially abundant taxa, acknowledging that many bacterial and fungal taxa remain uncharacterized with trait values that remain difficult to infer with a high degree of confidence.

**Fig 4:**
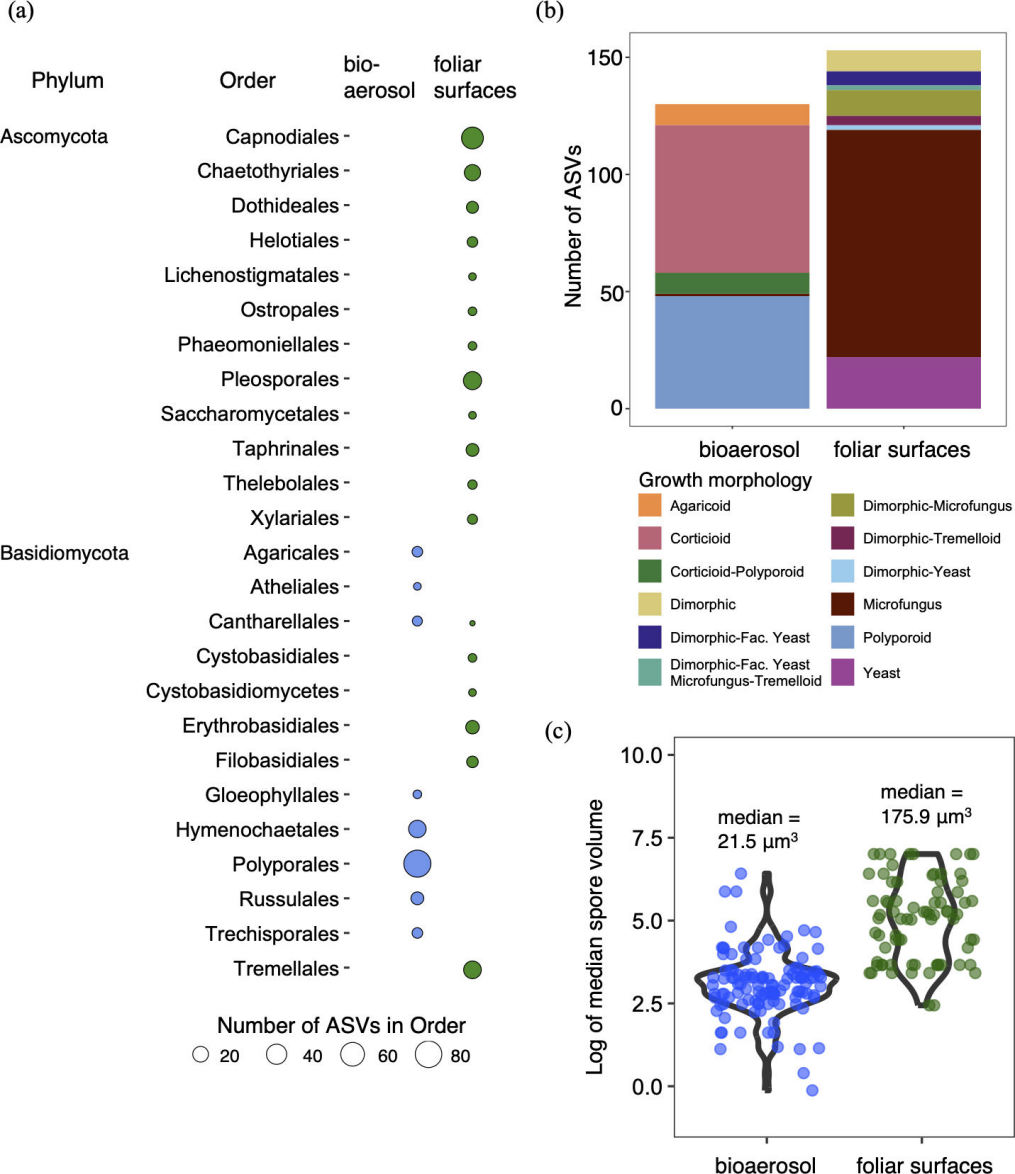
Taxonomy and traits of fungi enriched in bioaerosols vs the foliar surface source environment. Panel **a** depicts the taxonomic orders of the fungal amplicon sequence variants (ASVs) that were differentially abundant between bioaerosol and foliar surface samples. Only fungal orders with at least two representative ASVs are shown for clarity (representing 142/152 and 201/243 ASVs that were more abundant in bioaerosol or foliar surface samples, respectively). Hymenochaetales in panel a represents an *Incertae sedis* order within the Hymenochaetales class. Panels **b** and **c** show traits for the differentially abundant ASVs represented in panel a. Panel **b** illustrates the growth morphologies for the differentially abundant ASVs, as available in the FUNGuild database (73). In this panel’s legend, “fac.” is an abbreviation of “facultative.” Note that panels b and c have fewer numbers of ASVs than panel a because not all ASVs were represented in the respective trait databases. Panel **c** shows the uninucleate sexual spore volumes of fungal ASVs enriched in bioaerosol and foliar surface samples based on volumes in Aguilar-Trigueros et al. (72) (see Table S7 for a list of these spore size matches by fungal order).

We hypothesized that the fungal taxa detected in the air would be more likely to belong to groups that produce fruiting bodies because of the role that these specialized structures play as sites of production and release of spores (e.g., 34). Taxonomic patterns and available trait data provided clear support for this hypothesis. The clear majority of the 152 taxa enriched in the near-surface atmosphere relative to foliar surfaces belonged to the orders Polyporales (55.3% of bioaerosol-enriched ASVs), Hymenochaetales (17.8%), and Russulales (7.2%) (Fig. 4a). These orders are morphologically variable but include many members that produce aboveground fruiting bodies (91–94). Accordingly, of the 130 out of 152 bioaerosol-enriched taxa with data in FUNGuild (73), the majority were characterized as having fruiting body growth morphologies, most notably corticioid (48.5% of all bioaerosol-enriched ASVs with morphology data) and polyporoid (36.9%) morphologies (Fig. 4b). For example, there were five ASVs within *Trametes*, a genus known to produce large, recognizable shelf-like polyporoid fruiting bodies on living or dead wood (95), that were enriched in the air relative to foliar surfaces and that were present in 90% of bioaerosol samples. In contrast, the most abundant and ubiquitous group of fungi on the sampled foliar surfaces were members of the order Capnodiales (20.2% of the 243 foliar surface-enriched ASVs, Fig. 4a) that do not generally produce fruiting bodies (96). Of the 156 out of 243 of foliar surface-enriched ASVs with information in FUNGuild, most were either microfungi (62.2%) or yeasts (14.1%) (Fig. 4b). Our results align with previous hypotheses that the production of aboveground fruiting bodies is often associated with the capacity for longer distance dispersal through the air (97).

We next evaluated our hypothesis that species with smaller fungal spores would be more common in the near-surface atmosphere than on foliar surfaces. To do so, we used the database from Aguilar‐Trigueros et al. (72) to infer sexual spore volume data for bioaerosol- and foliar surface-associated taxa (see Materials and Methods). Spore volume data were available for 76.3% and 31.3% of bioaerosol-associated and foliar surface-associated ASVs, respectively, and these ASVs represented a range of fungal orders in both groups (Table S7). In support of our hypothesis, we found that fungi enriched in the bioaerosol samples had smaller sexual spores than did those more abundant on foliar surfaces (*W* = 818, *P* < 0.001), with a median of only 21.5 μm^3^ in the air compared to 175.9 μm^3^ on foliar surfaces (Fig. 4c). Importantly, bioaerosol-associated fungi still had smaller spores even if we considered the subsetted spore volume data set where we restricted our analysis only to ASVs for which there was minimal taxon-specific variation in spore volumes (*W* = 142, *P* < 0.01, Table S7). Previous modeling work suggests that, due to interactions between spore size, aerodynamics, and gravitational deposition, smaller propagules generally disperse farther than larger ones (35, 98). Therefore, smaller-sized spores may be selected for in contexts where more distant dispersal through the air is advantageous (90). For example, saprotrophic fungi—such as the members of Polyporales and Hymenochaetales (Fig. 4a, Table S7)—may produce numerous small spores to move among ephemeral or patchy substrates via aerial dispersal (90, 99). We note that we were unable to consider seasonality in spore release (7, 90, 100) or plasticity in spore size (e.g., 101). Future work should consider using microscopy to assess spore sizes across different seasons and locations to test the generality of our finding that fungi with smaller spores are more likely to be found in the atmosphere.

**Fig 5:**
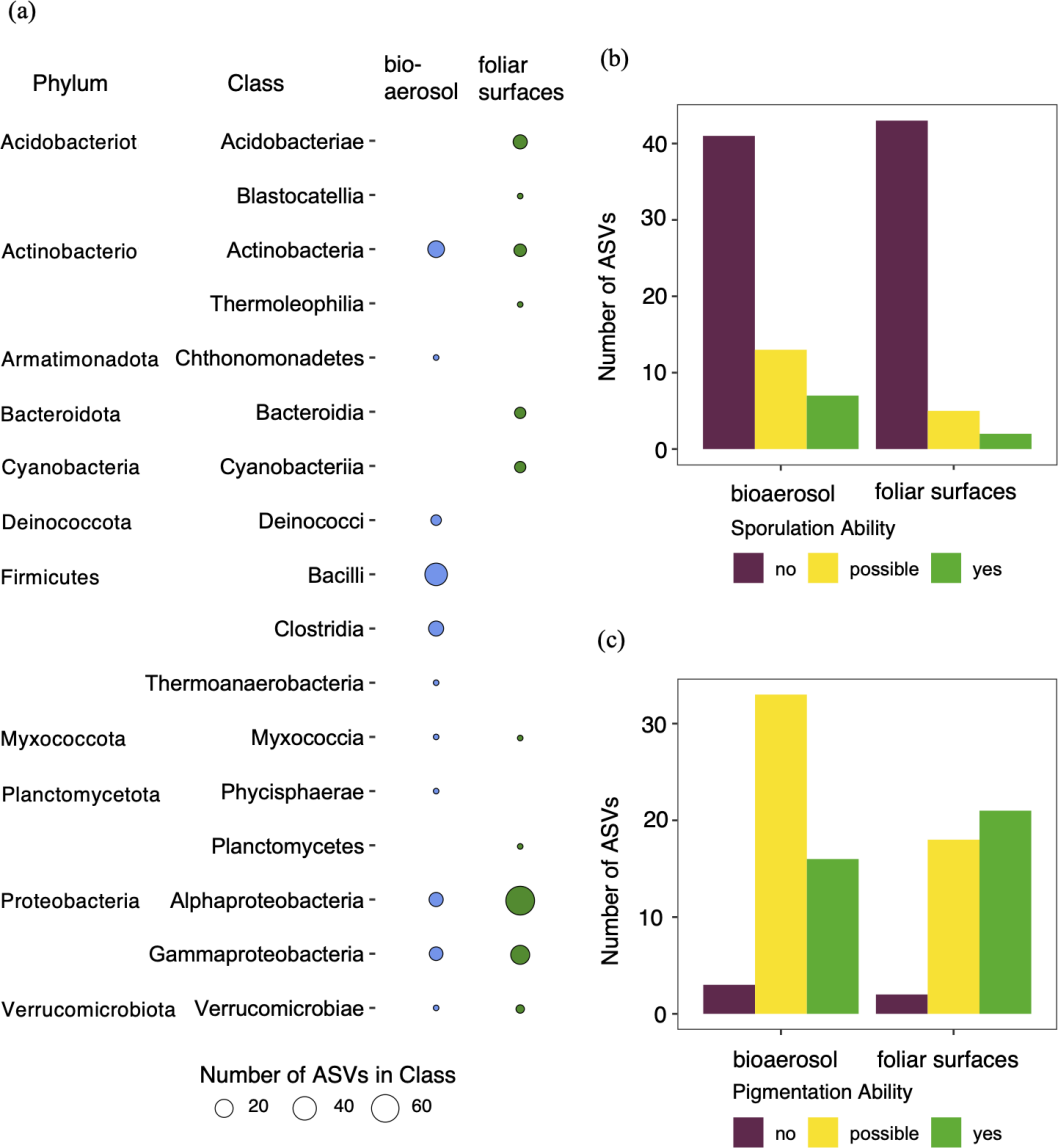
Taxonomy and traits of bacteria enriched in bioaerosols vs the foliar surface source environment. Panel **a** depicts the taxonomic classes of the bacterial amplicon sequence variants (ASVs) that were differentially abundant between bioaerosol and foliar surface samples, where 92/94 and 123/126 ASVs more abundant in bioaerosol or foliar surface samples, respectively, were assigned to class and are shown. Panels **b** and **c** show traits for the differentially abundant ASVs represented in panel a. Panel b shows the probable sporulation ability of each differentially abundant bacterial ASV, whereas panel c shows the probable pigmentation abilities of these taxa based on information available in the Madin et al. (75) and Barberán et al. (76) databases, respectively. “Possible” indicates that some taxonomic matches to a particular ASV could sporulate or produce pigment, but not all. Note that panels b and c have fewer numbers of ASVs than panel a because not all ASVs were represented in the respective trait databases.

We next hypothesized that bacteria which are capable of producing pigments and spores are more likely to be enriched in the bioaerosol samples compared to the foliar surface samples, following previous work demonstrating that these traits can promote survival of bacteria under stressful conditions typical of the atmospheric environment (reviewed in reference 2), including UV exposures and desiccation (36, 38, 102). To test these hypotheses, we first identified bacterial ASVs that were differentially abundant between bioaerosols (*n* = 94 ASVs) and the putative foliar surface source (*n* = 126 ASVs) (Fig. 5a). The top bacterial classes that were enriched in the near surface atmosphere included Bacilli (38.3% of ASVs), Actinobacteria (17.0%), and Clostridia (12.8%). For foliar surfaces, we found that the most abundant classes were Alphaproteobacteria (53.2% of ASVs), Gammaproteobacteria (18.3%), and Acidobacteriae (7.9%).

First, bacteria enriched in the air were nearly three times more likely to be capable of spore formation than were bacteria enriched in the foliar surface source (Fisher’s exact test, *P* < 0.05, odds ratio = 2.97, Fig. 5b). This is most evident given the notable abundance of members of the class Bacilli among the taxa enriched in bioaerosol samples (Fig. 5a), as many members of this group are known to produce endospores that are highly resistant to environmental stressors (e.g., 102). Our result is consistent with a metagenomics-based study which identified a higher abundance of sporulation genes in dust microbiome samples than in distantly collected soils (30). Interestingly, two of our study’s putative spore-formers ASV 48 (*Cystobacter* sp.) and ASV 395 (*Pseudomonas* sp.) were among the most abundant ASVs in bioaerosol samples but were not detected in our soil and foliar surface samples (Table S5), indicating the possibility that they were aerosolized and traveled from a more distant source outside of our experimental units. By comparing bioaerosol samples and putative local sources, our study provides evidence supporting the longstanding idea that spores originating in terrestrial environments may be adapted for transport through the near-surface atmosphere where environmental conditions can limit bacterial survival (1, 8). In contrast, we found that the bacterial taxa enriched in the bioaerosol samples were not more likely to be pigment-producing than foliar surface-associated bacteria (Fisher’s exact test, *P* = 1, odds ratio = 0.84, Fig. 5c). Instead, both environments were characterized by relatively high numbers of taxa that might produce pigments (Fig. 5c). A possible explanation for this unexpected result is that, because bacterial cells on the surface of plant leaves also often contend with high UV stressors (103), the selection pressure for pigmentation is similar in the near-surface atmosphere and foliar surface source environment. We note that for both bacterial sporulation and pigmentation, our study was constrained by the availability of trait data, a common limitation in studies of environmental microbiomes (104, 105). Although we were able to match ~50% of differentially abundant ASVs for our sporulation analysis and ~42% of ASVs with pigmentation information, it is possible that the observed trait patterns would be different if corresponding trait data were available for a broader diversity of the bacterial taxa detected.

### Conclusion

The near-surface atmosphere contains diverse assemblages of bacteria and fungi that are distinct from terrestrial environments, but the relationships between the aeromicrobiome and the terrestrial sources of microbes to the atmosphere are rarely examined directly. We quantified the relative contributions of fungi and bacteria from local leaves and soils to the aeromicrobiome, revealing that foliar surfaces are the more important source of microbes to the near-surface atmosphere at our site. Yet, despite the clear signature of foliar-surface associated microbes in bioaerosols, the bioaerosols collected from the adjacent open savanna-like patch and forested matrix habitats did not consistently differ with respect to the amounts or types of microbial taxa. Considered alongside previous work reporting that bioaerosol composition varies across land use types at larger spatial scales (10, 16–19), the homogenization of the aeromicrobiome at the smaller spatial scales studied here highlights the importance of future work focused on quantifying the spatial scale at which differences in source environments lead to corresponding differences in aeromicrobiomes and how these differences are modulated by changes in atmospheric conditions. Finally, our comparison of microbes enriched in bioaerosols and the foliar surface source environment provided evidence that the near-surface atmosphere is enriched in fungal taxa with aboveground fruiting bodies and that produce smaller sexual spores and in bacteria capable of undergoing sporulation. Identifying these traits represents an important step in building a more predictive understanding of aeromicrobiome dynamics and the differential capacity for aerial dispersal across a broad diversity of microbial taxa.

## Data Availability

Sequence data are publicly available via the National Center for Biotechnology Information (NCBI) Sequence Read Archive (SRA; BioProject ID: PRJNA1263026). All analyses were performed using publicly available statistical packages and data, all of which are cited within the paper. Code used for analyses can be found at https://github.com/clairecwinfrey/BioaerosolSourcesTraits.

## SUPPLEMENTAL MATERIAL

Supplemental material aem.02348-25-s0001.docx Tables S1 to S7; Fig. S1 to S4.

ASM does not own the copyrights to Supplemental Material that may be linked to, or accessed through, an article. The authors have granted ASM a non-exclusive, world-wide license to publish the Supplemental Material files. Please contact the corresponding author directly for reuse.
