# Supplementary material for: Sources and traits of bacteria and fungi found in the near-surface atmosphere
Source: Appl Environ Microbiol. 2026 Jul 2;92(7):e02348-25. doi: 10.1128/aem.02348-25 (PMC13390485; doi:10.1128/aem.02348-25)
Supplement: Supplemental material — Tables S1 to S7; Fig. S1 to S4. [file aem.02348-25-s0001.docx]

**The sources and traits of bacteria and fungi found in the near-surface atmosphere**

Supplementary Information

**Table S1.** Metadata and number of gene copies associated with each bioaerosol sample. ID represents the sample ID. EU represents the replicate “experimental unit” where the sample was collected. Weather variables are given for the 24-hour sampling period, based on measurements every 15 minutes. Wind data was measured in the center of each EU at a height of 0.91 m. Temperature, humidity, and total rainfall (per sampling day) were measured by the Savannah River National Lab Atmospheric Technologies Group near the center of the Savannah River Site from a height of 2 m. Numbers of ITS rRNA gene (fungi) and 16S rRNA gene (bacteria) copy numbers are expressed per meter cubed of sampled air. NAs represent samples that did not have enough reads to meet the rarefying threshold or that did not amplify during quantitative PCR.

| ID | EU | Habitat | Start  Date | Mean  wind  speed  (km/  hour) | Mean  wind  gust  speed  (km/  hour) | Mean  temp.  °C | High temp.  °C | Low temp.  °C | Mean relative humi-dity  (%) | Rain-fall  (cm) | ITS  rRNA gene  copies | 16S  rRNA  gene  copies |
| --- | --- | --- | --- | --- | --- | --- | --- | --- | --- | --- | --- | --- |
| 1 | 52 | open patch | 16-Jun-22 | 0.6 | 5.1 | 28.2 | 36.9 | 22.0 | 65.8 | 0.9 | 2019590 | 20541 |
| 2 | 52 | open patch | 16-Jun-22 | 0.6 | 5.1 | 28.2 | 36.9 | 22.0 | 65.8 | 0.9 | 3327291 | 39966 |
| 4 | 54S | forested matrix | 8-Jul-22 | 1.1 | 6.1 | 25.9 | 33.4 | 23.3 | 86.0 | 0.5 | 2659937 | 78856 |
| 9 | 53S | forested matrix | 6-Jul-22 | 1.2 | 4.9 | 28.0 | 35.2 | 23.7 | 73.2 | 1.5 | 5361675 | 12557 |
| 10 | 8 | forested matrix | 4-Jul-22 | 0.6 | 3.7 | 27.7 | 34.0 | 22.6 | 81.4 | 0.0 | 5036732 | NA |
| 14 | 53S | open patch | 6-Jul-22 | 1.2 | 4.9 | 28.0 | 35.2 | 23.7 | 73.2 | 1.5 | 4049481 | NA |
| 15 | 54S | open patch | 30-Jun-22 | 0.9 | 4.1 | 25.1 | 31.2 | 21.8 | 82.5 | 1.4 | 9349617 | NA |
| 16 | 53S | forested matrix | 6-Jul-22 | 1.2 | 4.9 | 28.0 | 35.2 | 23.7 | 73.2 | 1.5 | 7655828 | NA |
| 17 | 52 | forested matrix | 1-Jul-22 | 0.3 | 2.4 | 25.7 | 34.6 | 21.0 | 82.1 | 0.0 | 23503602 | NA |
| 18 | 54S | open patch | 24-Jun-22 | 1.3 | 5.6 | 26.9 | 33.1 | 21.2 | 66.5 | 0.9 | 2394735 | NA |
| 19 | 53S | forested matrix | 27-Jun-22 | 0.1 | 1.5 | 24.9 | 30.8 | 23.0 | 74.9 | 1.1 | 660402 | NA |
| 20 | 53S | open patch | 6-Jul-22 | 1.2 | 4.9 | 28.0 | 35.2 | 23.7 | 73.2 | 1.5 | 3442126 | 24749 |
| 23 | 54S | forested matrix | 24-Jun-22 | 1.3 | 5.6 | 26.9 | 33.1 | 21.2 | 66.5 | 0.9 | 1153470 | 1387 |
| 25 | 8 | open patch | 20-Jun-22 | 1.0 | 4.6 | 26.2 | 35.0 | 17.6 | 51.8 | 0.0 | 812608 | NA |
| 28 | 52 | forested matrix | 18-Jun-22 | 1.0 | 5.8 | 25.1 | 32.3 | 19.1 | 38.0 | 0.0 | 5159259 | 12903 |
| 30 | 53S | forested matrix | 27-Jun-22 | 0.1 | 1.5 | 24.9 | 30.8 | 23.0 | 74.9 | 1.1 | 2705640 | 5832 |
| 31 | 54S | forested matrix | 8-Jul-22 | 1.1 | 6.1 | 25.9 | 33.4 | 23.3 | 86.0 | 0.5 | 6507056 | 7682 |
| 33 | 53S | open patch | 22-Jun-22 | 1.1 | 4.6 | 28.2 | 37.1 | 22.0 | 48.3 | 0.3 | 4689790 | 1708 |
| 34 | 8 | open patch | 23-Jun-22 | 1.3 | 7.1 | 27.4 | 36.9 | 21.5 | 75.5 | 0.2 | 4966076 | 6028 |
| 35 | 53S | open patch | 6-Jul-22 | 1.2 | 4.9 | 28.0 | 35.2 | 23.7 | 73.2 | 1.5 | 522762 | NA |
| 36 | 53S | forested matrix | 22-Jun-22 | 1.1 | 4.6 | 28.2 | 37.1 | 22.0 | 48.3 | 0.3 | 5369841 | 52038 |
| 38 | 8 | open patch | 23-Jun-22 | 1.3 | 7.1 | 27.4 | 36.9 | 21.5 | 75.5 | 0.2 | 4166061 | 39814 |
| 39 | 52 | open patch | 18-Jun-22 | 1.0 | 5.8 | 25.1 | 32.3 | 19.1 | 38.0 | 0.0 | 3826832 | 39644 |
| 44 | 54S | forested matrix | 5-Jul-22 | 1.0 | 5.2 | 27.9 | 35.2 | 23.7 | 77.2 | 0.0 | 15257733 | 25749 |
| 45 | 53S | forested matrix | 2-Jul-22 | 0.8 | 4.2 | 27.2 | 34.3 | 21.9 | 76.8 | 0.0 | 9449940 | 25673 |
| 46 | 8 | open patch | 4-Jul-22 | 0.6 | 3.7 | 27.7 | 34.0 | 22.6 | 81.4 | 0.0 | 3220577 | 150899 |
| 47 | 52 | forested matrix | 16-Jun-22 | 0.6 | 5.1 | 28.2 | 36.9 | 22.0 | 65.8 | 0.9 | 2098064 | 4670 |
| 48 | 53S | open patch | 2-Jul-22 | 0.8 | 4.2 | 27.2 | 34.3 | 21.9 | 76.8 | 0.0 | 2275311 | 4972 |
| 49 | 54S | forested matrix | 5-Jul-22 | 1.0 | 5.2 | 27.9 | 35.2 | 23.7 | 77.2 | 0.0 | 3259536 | 6231 |
| 50 | 54S | forested matrix | 5-Jul-22 | 1.0 | 5.2 | 27.9 | 35.2 | 23.7 | 77.2 | 0.0 | 2963374 | 4123 |
| 51 | 54S | open patch | 24-Jun-22 | 1.3 | 5.6 | 26.9 | 33.1 | 21.2 | 66.5 | 0.9 | 2330396 | 4422 |
| 54 | 8 | forested matrix | 20-Jun-22 | 1.0 | 4.6 | 26.2 | 35.0 | 17.6 | 51.8 | 0.0 | 2973981 | 4792 |
| 56 | 53S | forested matrix | 6-Jul-22 | 1.2 | 4.9 | 28.0 | 35.2 | 23.7 | 73.2 | 1.5 | 5440735 | 10391 |
| 60 | 52 | forested matrix | 18-Jun-22 | 1.0 | 5.8 | 25.1 | 32.3 | 19.1 | 38.0 | 0.0 | 1206642 | 89035 |
| 61 | 8 | forested matrix | 4-Jul-22 | 0.6 | 3.7 | 27.7 | 34.0 | 22.6 | 81.4 | 0.0 | 4007835 | 7888 |
| 62 | 54S | forested matrix | 30-Jun-22 | 0.9 | 4.1 | 25.1 | 31.2 | 21.8 | 82.5 | 1.4 | 4812344 | 9522 |
| 63 | 54S | open patch | 5-Jul-22 | 1.0 | 5.2 | 27.9 | 35.2 | 23.7 | 77.2 | 0.0 | 4763367 | 13233 |
| 64 | 53S | forested matrix | 2-Jul-22 | 0.8 | 4.2 | 27.2 | 34.3 | 21.9 | 76.8 | 0.0 | 8201962 | 7416 |
| 65 | 54S | open patch | 5-Jul-22 | 1.0 | 5.2 | 27.9 | 35.2 | 23.7 | 77.2 | 0.0 | 6812768 | 22740 |
| 66 | 54S | forested matrix | 30-Jun-22 | 0.9 | 4.1 | 25.1 | 31.2 | 21.8 | 82.5 | 1.4 | 1712915 | 29840 |
| 67 | 52 | forested matrix | 1-Jul-22 | 0.3 | 2.4 | 25.7 | 34.6 | 21.0 | 82.1 | 0.0 | 5308662 | 120529 |
| 69 | 53S | open patch | 27-Jun-22 | 0.1 | 1.5 | 24.9 | 30.8 | 23.0 | 74.9 | 1.1 | 374267 | 25349 |
| 70 | 8 | forested matrix | 20-Jun-22 | 1.0 | 4.6 | 26.2 | 35.0 | 17.6 | 51.8 | 0.0 | 1832840 | 87813 |
| 71 | 52 | forested matrix | 18-Jun-22 | 1.0 | 5.8 | 25.1 | 32.3 | 19.1 | 38.0 | 0.0 | 2468563 | 176607 |
| 72 | 8 | forested matrix | 20-Jun-22 | 1.0 | 4.6 | 26.2 | 35.0 | 17.6 | 51.8 | 0.0 | 708342 | 11778 |
| 76 | 8 | forested matrix | 29-Jun-22 | 0.5 | 3.7 | 23.3 | 31.8 | 20.9 | 90.6 | 6.7 | 12305126 | NA |
| 79 | 53S | open patch | 6-Jul-22 | 1.2 | 4.9 | 28.0 | 35.2 | 23.7 | 73.2 | 1.5 | 5470069 | 50062 |
| 81 | 52 | forested matrix | 26-Jun-22 | 0.4 | 3.6 | 26.7 | 33.2 | 20.0 | 60.0 | 0.0 | 1473320 | NA |
| 83 | 8 | open patch | 29-Jun-22 | 0.5 | 3.7 | 23.3 | 31.8 | 20.9 | 90.6 | 6.7 | 3476633 | 17357 |
| 84 | 53S | forested matrix | 6-Jul-22 | 1.2 | 4.9 | 28.0 | 35.2 | 23.7 | 73.2 | 1.5 | 1307230 | NA |
| 85 | 8 | forested matrix | 29-Jun-22 | 0.5 | 3.7 | 23.3 | 31.8 | 20.9 | 90.6 | 6.7 | 5639792 | 88150 |
| 86 | 52 | open patch | 18-Jun-22 | 1.0 | 5.8 | 25.1 | 32.3 | 19.1 | 38.0 | 0.0 | 1812200 | 50777 |
| 88 | 8 | forested matrix | 29-Jun-22 | 0.5 | 3.7 | 23.3 | 31.8 | 20.9 | 90.6 | 6.7 | 2456747 | 29063 |
| 89 | 54S | open patch | 8-Jul-22 | 1.1 | 6.1 | 25.9 | 33.4 | 23.3 | 86.0 | 0.5 | 4707819 | 56005 |
| 90 | 52 | forested matrix | 18-Jun-22 | 1.0 | 5.8 | 25.1 | 32.3 | 19.1 | 38.0 | 0.0 | 2240371 | 25991 |
| 91 | 8 | open patch | 20-Jun-22 | 1.0 | 4.6 | 26.2 | 35.0 | 17.6 | 51.8 | 0.0 | 566536 | 13484 |
| 92 | 54S | open patch | 8-Jul-22 | 1.1 | 6.1 | 25.9 | 33.4 | 23.3 | 86.0 | 0.5 | 14687403 | NA |
| 93 | 54S | forested matrix | 8-Jul-22 | 1.1 | 6.1 | 25.9 | 33.4 | 23.3 | 86.0 | 0.5 | 5975501 | 237754 |
| 94 | 52 | open patch | 26-Jun-22 | 0.4 | 3.6 | 26.7 | 33.2 | 20.0 | 60.0 | 0.0 | 482385 | 26523 |
| 95 | 52 | open patch | 26-Jun-22 | 0.4 | 3.6 | 26.7 | 33.2 | 20.0 | 60.0 | 0.0 | 866079 | 21316 |
| 96 | 52 | forested matrix | 16-Jun-22 | 0.6 | 5.1 | 28.2 | 36.9 | 22.0 | 65.8 | 0.9 | 459873 | 6643 |
| 97 | 53S | open patch | 27-Jun-22 | 0.1 | 1.5 | 24.9 | 30.8 | 23.0 | 74.9 | 1.1 | 3002076 | 12677 |
| 99 | 54S | forested matrix | 24-Jun-22 | 1.3 | 5.6 | 26.9 | 33.1 | 21.2 | 66.5 | 0.9 | 147425 | NA |
| 100 | 8 | forested matrix | 20-Jun-22 | 1.0 | 4.6 | 26.2 | 35.0 | 17.6 | 51.8 | 0.0 | 577148 | 15364 |
| 103 | 53S | forested matrix | 2-Jul-22 | 0.8 | 4.2 | 27.2 | 34.3 | 21.9 | 76.8 | 0.0 | 3414902 | 220359 |
| 105 | 53S | forested matrix | 27-Jun-22 | 0.1 | 1.5 | 24.9 | 30.8 | 23.0 | 74.9 | 1.1 | 306433 | 38242 |
| 106 | 52 | forested matrix | 26-Jun-22 | 0.4 | 3.6 | 26.7 | 33.2 | 20.0 | 60.0 | 0.0 | 629915 | 23720 |
| 108 | 8 | open patch | 20-Jun-22 | 1.0 | 4.6 | 26.2 | 35.0 | 17.6 | 51.8 | 0.0 | 5254918 | 30607 |
| 109 | 8 | forested matrix | 29-Jun-22 | 0.5 | 3.7 | 23.3 | 31.8 | 20.9 | 90.6 | 6.7 | 12818873 | 241716 |
| 110 | 8 | open patch | 4-Jul-22 | 0.6 | 3.7 | 27.7 | 34.0 | 22.6 | 81.4 | 0.0 | 7619258 | 176397 |
| 111 | 53S | forested matrix | 2-Jul-22 | 0.8 | 4.2 | 27.2 | 34.3 | 21.9 | 76.8 | 0.0 | 971651 | 11536 |
| 112 | 54S | forested matrix | 24-Jun-22 | 1.3 | 5.6 | 26.9 | 33.1 | 21.2 | 66.5 | 0.9 | 2250969 | NA |
| 113 | 52 | open patch | 1-Jul-22 | 0.3 | 2.4 | 25.7 | 34.6 | 21.0 | 82.1 | 0.0 | 58624006 | NA |
| 115 | 52 | forested matrix | 1-Jul-22 | 0.3 | 2.4 | 25.7 | 34.6 | 21.0 | 82.1 | 0.0 | 35193509 | NA |
| 116 | 52 | open patch | 1-Jul-22 | 0.3 | 2.4 | 25.7 | 34.6 | 21.0 | 82.1 | 0.0 | 28196393 | 43012 |
| 117 | 53S | open patch | 2-Jul-22 | 0.8 | 4.2 | 27.2 | 34.3 | 21.9 | 76.8 | 0.0 | 751274 | NA |
| 118 | 54S | open patch | 30-Jun-22 | 0.9 | 4.1 | 25.1 | 31.2 | 21.8 | 82.5 | 1.4 | 5326380 | 28788 |
| 119 | 8 | open patch | 4-Jul-22 | 0.6 | 3.7 | 27.7 | 34.0 | 22.6 | 81.4 | 0.0 | 11723853 | 215602 |
| 120 | 8 | open patch | 29-Jun-22 | 0.5 | 3.7 | 23.3 | 31.8 | 20.9 | 90.6 | 6.7 | 7724319 | 57112 |
| 121 | 54S | open patch | 30-Jun-22 | 0.9 | 4.1 | 25.1 | 31.2 | 21.8 | 82.5 | 1.4 | 19862281 | 77988 |
| 124 | 8 | forested matrix | 4-Jul-22 | 0.6 | 3.7 | 27.7 | 34.0 | 22.6 | 81.4 | 0.0 | 12271181 | NA |
| 125 | 52 | open patch | 1-Jul-22 | 0.3 | 2.4 | 25.7 | 34.6 | 21.0 | 82.1 | 0.0 | 15692720 | NA |
| 127 | 52 | forested matrix | 26-Jun-22 | 0.4 | 3.6 | 26.7 | 33.2 | 20.0 | 60.0 | 0.0 | 876095 | 48706 |
| 128 | 8 | forested matrix | 23-Jun-22 | 1.3 | 7.1 | 27.4 | 36.9 | 21.5 | 75.5 | 0.2 | 2811613 | 365440 |
| 129 | 52 | forested matrix | 1-Jul-22 | 0.3 | 2.4 | 25.7 | 34.6 | 21.0 | 82.1 | 0.0 | 586185 | NA |
| 130 | 54S | open patch | 30-Jun-22 | 0.9 | 4.1 | 25.1 | 31.2 | 21.8 | 82.5 | 1.4 | 6268123 | 49061 |
| 131 | 8 | forested matrix | 23-Jun-22 | 1.3 | 7.1 | 27.4 | 36.9 | 21.5 | 75.5 | 0.2 | 2089954 | 124467 |
| 132 | 53S | open patch | 22-Jun-22 | 1.1 | 4.6 | 28.2 | 37.1 | 22.0 | 48.3 | 0.3 | 3907047 | 22515 |
| 133 | 52 | open patch | 1-Jul-22 | 0.3 | 2.4 | 25.7 | 34.6 | 21.0 | 82.1 | 0.0 | 15279985 | NA |
| 134 | 53S | forested matrix | 22-Jun-22 | 1.1 | 4.6 | 28.2 | 37.1 | 22.0 | 48.3 | 0.3 | 2021136 | 57472 |
| 135 | 54S | forested matrix | 30-Jun-22 | 0.9 | 4.1 | 25.1 | 31.2 | 21.8 | 82.5 | 1.4 | 1626585 | 57516 |
| 136 | 53S | open patch | 2-Jul-22 | 0.8 | 4.2 | 27.2 | 34.3 | 21.9 | 76.8 | 0.0 | 547320 | NA |
| 139 | 54S | forested matrix | 30-Jun-22 | 0.9 | 4.1 | 25.1 | 31.2 | 21.8 | 82.5 | 1.4 | 4665424 | 121179 |
| 140 | 54S | forested matrix | 8-Jul-22 | 1.1 | 6.1 | 25.9 | 33.4 | 23.3 | 86.0 | 0.5 | 11320108 | NA |
| 141 | 8 | forested matrix | 4-Jul-22 | 0.6 | 3.7 | 27.7 | 34.0 | 22.6 | 81.4 | 0.0 | 10708101 | 25017 |
| 142 | 54S | open patch | 8-Jul-22 | 1.1 | 6.1 | 25.9 | 33.4 | 23.3 | 86.0 | 0.5 | 15998281 | 87678 |
| 143 | 52 | open patch | 26-Jun-22 | 0.4 | 3.6 | 26.7 | 33.2 | 20.0 | 60.0 | 0.0 | 1681931 | 39215 |
| 144 | 54S | forested matrix | 24-Jun-22 | 1.3 | 5.6 | 26.9 | 33.1 | 21.2 | 66.5 | 0.9 | 199531 | 5870 |
| 145 | 53S | forested matrix | 22-Jun-22 | 1.1 | 4.6 | 28.2 | 37.1 | 22.0 | 48.3 | 0.3 | 379788 | 36266 |
| 146 | 52 | open patch | 26-Jun-22 | 0.4 | 3.6 | 26.7 | 33.2 | 20.0 | 60.0 | 0.0 | 364250 | NA |
| 147 | 54S | open patch | 24-Jun-22 | 1.3 | 5.6 | 26.9 | 33.1 | 21.2 | 66.5 | 0.9 | 88180 | 15427 |
| 148 | 53S | open patch | 2-Jul-22 | 0.8 | 4.2 | 27.2 | 34.3 | 21.9 | 76.8 | 0.0 | 4570283 | NA |
| 149 | 54S | forested matrix | 5-Jul-22 | 1.0 | 5.2 | 27.9 | 35.2 | 23.7 | 77.2 | 0.0 | 5409442 | 79222 |
| 150 | 52 | open patch | 18-Jun-22 | 1.0 | 5.8 | 25.1 | 32.3 | 19.1 | 38.0 | 0.0 | 4161495 | 73526 |
| 152 | 52 | open patch | 16-Jun-22 | 0.6 | 5.1 | 28.2 | 36.9 | 22.0 | 65.8 | 0.9 | 1075951 | 8830 |
| 153 | 53S | forested matrix | 22-Jun-22 | 1.1 | 4.6 | 28.2 | 37.1 | 22.0 | 48.3 | 0.3 | 4329382 | 23515 |
| 154 | 8 | open patch | 29-Jun-22 | 0.5 | 3.7 | 23.3 | 31.8 | 20.9 | 90.6 | 6.7 | 6962281 | 107648 |
| 155 | 8 | open patch | 4-Jul-22 | 0.6 | 3.7 | 27.7 | 34.0 | 22.6 | 81.4 | 0.0 | 3109693 | 27447 |
| 156 | 8 | open patch | 23-Jun-22 | 1.3 | 7.1 | 27.4 | 36.9 | 21.5 | 75.5 | 0.2 | 6445260 | 104453 |
| 158 | 8 | forested matrix | 23-Jun-22 | 1.3 | 7.1 | 27.4 | 36.9 | 21.5 | 75.5 | 0.2 | 2038525 | 74942 |

**Table S2.** Species of plants sampled for their foliar surface microbial communities, arranged by experimental unit (EU) that they were collected in. Note that many species were sampled in multiple EUs. EUPCOM sampled in EU 52 returned too few 16S rRNA reads to be considered in our bacterial analyses.

| **EU** | **Plant ID** | **Genus** | **Species** | **Common Name** |
| --- | --- | --- | --- | --- |
| 8 | ANDSPP | *Andropogon* | unknown | bluestem |
| 8 | DICACI | *Dichanthelium* | *aciculare* | needleleaf rosette grass |
| 8 | DICVOL | *Dichanthelium* | *villosissimum* | white-haired witchgrass |
| 8 | DIOVIR | *Diospyros* | *virginiana* | common persimmon |
| 8 | PINPAL | *Pinus* | *palustris* | long-leaf pine |
| 8 | PINTAE | *Pinus* | *taeda* | loblolly pine |
| 8 | PITGRA | *Pityopsis* | *graminifolia* | narrow-leaf silkgrass |
| 8 | PRUSER | *Prunus* | *serotina* | black cherry |
| 8 | QUEHEM | *Quercus* | *hemisphaerica* | sand laurel oak |
| 8 | QUENIG | *Quercus* | *nigra* | water oak |
| 8 | RHUCOP | *Rhus* | *copallinum* | winged sumac |
| 8 | SOLNEM | *Solidago* | *nemoralis* | gray goldenrod |
| 8 | VACARB | *Vaccinium* | *arboreum* | sparkleberry |
| 8 | VACSTA | *Vaccinium* | *stamineum* | deerberry |
| 8 | VITROT | *Vitis* | *rotundafolia* | muscadine grape |
| 52 | ANDSPP | *Andropogon* | *unknown* | bluestem |
| 52 | ASTTOR | *Astor* | *tortefolius* | dixie white-topped aster |
| 52 | CARPAL | *Carya* | *pallida* | sand hickory |
| 52 | EUPCOM | *Eupatorium* | *compositifolium* | yankeeweed |
| 52 | GELSEM | *Gelsemium* | *sempervirens* | Carolina jessamine |
| 52 | GYMAMB | *Gymnopogon* | *ambiguus* | bearded skeletongrass |
| 52 | LIQSTY | *Liquidambar* | *styraciflua* | American sweetgum |
| 52 | PINTAE | *Pinus* | *taeda* | loblolly pine |
| 52 | PRUSER | *Prunus* | *serotina* | black cherry |
| 52 | QUEINC | *Quercus* | *incana* | bluejack oak |
| 52 | QUEMAG | *Quercus* | *margarettae* | sand post oak |
| 52 | QUENIG | *Quercus* | *nigra* | water oak |
| 52 | RHUCOP | *Rhus* | *copallinum* | winged sumac |
| 52 | SOLODO | *Solidago* | *odora* | licorice goldenrod |
| 52 | RHUTOX | *Toxicodendrom* | *pubescens* | poison oak |
| 52 | VACSTA | *Vacinium* | *stamineum* | deerberry |
| 52 | VITROT | *Vitis* | *rotundafolia* | muscadine grape |
| 53S | ANDSPP | *Andropogon* | *unknown* | bluestem |
| 53S | DIOVIR | *Diospyros* | *virginiana* | common persimmon |
| 53S | GELSEM | *Gelsemium* | *sempervirens* | Carolina jessamine |
| 53S | PINPAL | *Pinus* | *palustris* | long-leaf pine |
| 53S | PITGRA | *Pityopsis* | *graminifolia* | narrow-leaf silkgrass |
| 53S | QUENIG | *Quercus* | *nigra* | water oak |
| 53S | RHUCOP | *Rhus* | *copallinum* | winged sumac |
| 53S | ASTPAT | *Sericocarpus* | *asteroides* | white-topped aster |
| 53S | SOLNEM | *Solidago* | *nemoralis* | gray goldenrod |
| 53S | SORNUT | *Sorghastrum* | *nutans* | nodding indiangrass |
| 53S | VACSTA | *Vaccinium* | *stamineum* | deerberry |
| 53S | VITROT | *Vitis* | *rotundafolia* | muscadine grape |
| 54S | ANDSPP | *Andropogon* | *unknown* | bluestem |
| 54S | CENVIR | *Centrosema* | *virginanum* | spurred butterfly pea |
| 54S | DIOVIR | *Diospyros* | *virginiana* | common persimmon |
| 54S | EUPCUN | *Eupatorium* | *glaucescens* | waxy boneset |
| 54S | PINPAL | *Pinus* | *palustris* | long-leaf pine |
| 54S | PITGRA | *Pityopsis* | *graminifolia* | narrow-leaf silkgrass |
| 54S | PRUSER | *Prunus* | *serotina* | black cherry |
| 54S | QUEINC | *Quercus* | *incana* | bluejack oak |
| 54S | QUEMAG | *Quercus* | *margarettae* | sand post oak |
| 54S | RHUCOP | *Rhus* | *copallinum* | winged sumac |
| 54S | SASALB | *Sassafras* | *albidum* | sassafras |
| 54S | SOLODO | *Solidago* | *odora* | licorice goldenrod |
| 54S | RHUTOX | *Toxicodendrom* | *pubescens* | poison oak |
| 54S | VACSTA | *Vaccinium* | *stamineum* | deerberry |
| 54S | VITROT | *Vitis* | *rotundafolia* | muscadine grape |

Table S3. Amplicon sequence variants (ASVs) that were identified as lab contaminants from either bioaerosol sample or foliar surface sample processing. All identified contaminants were bacteria; no fungal ASVs were identified as lab contaminants. Median relative abundances and standard deviations of each contaminant in bioaerosol or foliar surface samples before removal of the ASV are shown. Samples included in the calculations are the 84 (out of 110) bioaerosol samples and 58 (out of 60) foliar surface samples retained after rarefying samples in 16S rRNA gene read dataset to 5,500 reads. After removing from further analysis any sample or blank with fewer than 5,500 16S rRNA gene reads, 8 out of 39 bioaerosol-associated 16S blanks remained (n = 6 field blanks, n = 2 wash buffer blanks) and 3 out of 9 foliar surface-associated 16S blanks remained (n = 3 foliar surface field blanks). For the ITS rRNA gene reads (not shown below because no fungal ASV as identified as a contaminant), we removed any sample or blank with fewer than 8,500 reads from further analysis. 2 out of 39 bioaerosol-associated ITS controls remained (n = 2 field blanks) and no foliar surface associated ITS blanks remained.

| **Type of contam-**  **inant** | **ASV name** | **Phylum** | **Class** | **Order** | **Family** | **Genus** | **Median**  **rel. abund.**  **% (SD)** |
| --- | --- | --- | --- | --- | --- | --- | --- |
| bioaerosol | 35 | Deino-coccota | Deinococci | Thermales | Thermaceae | *Thermus* | 0.16 (2.80) |
| bioaerosol | 79 | Firmicutes | Bacilli | Bacillales | Bacillaceae | *Geobacillus* | 0.13 (1.96) |
| bioaerosol | 88 | Firmicutes | Bacilli | Lactoba-cillales | Entero-coccaceae | *Tetragenococcus* | 0.10 (1.12) |
| bioaerosol | 110 | Proteo-bacteria | Gammaproteo-bacteria | Burkhol-deriales | Burkhol-deriaceae | *Burkholderia-Caballeronia-Paraburkholderia* | 0.00 (1.08) |
| bioaerosol | 147 | Firmicutes | Bacilli | Lactoba-cillales | Entero-coccaceae | *Tetragenococcus* | 0.02 (1.56) |
| bioaerosol | 1455 | Actino-bacteriota | Actinobacteria | Coryne-bacteriales | Nocard-  iaceae | *Rhodococcus* | 0.05 (0.03) |
| bioaerosol | 1528 | Deino-coccota | Deinococci | Thermales | Thermaceae | *Thermus* | 0.00 (0.11) |
| bioaerosol | 2292 | Proteo-bacteria | Gammaproteo-bacteria | Burkhol-deriales | Sulfuri-cellaceae | *Sulfuriferula* | 0.04 (0.03) |
| bioaerosol | 3029 | Firmicutes | Bacilli | Bacillales | Bacillaceae | *Geobacillus* | 0.00 (0.08) |
| bioaerosol | 3115 | Firmicutes | Bacilli | Lactoba-cillales | Entero-coccaceae | *Tetragenococcus* | 0.00 (0.04) |
| foliar surface | 11 | Proteobacteria | Alphaproteo-bacteria | Rhizobiales | Beijerinckiaceae | *Methylobacterium-Methylorubrum* | 1.93 (1.97) |
| foliar surface | 79 | Firmicutes | Bacilli | Bacillales | Bacillaceae | *Geobacillus* | 0.00 (0.02) |
| foliar surface | 88 | Firmicutes | Bacilli | Lactobacillales | Entero-coccaceae | *Tetragenococcus* | 0.00 (0.01) |
| foliar surface | 110 | Proteobacteria | Gammaproteo-bacteria | Burkholderiales | Burkhol-  deriaceae | *Burkholderia-Caballeronia-Paraburkholderia* | 0.01 (0.21) |
| foliar surface | 132 | Firmicutes | Bacilli | Staphylococcales | Staphylo-coccaceae | *Staphylococcus* | 0.00 (0.03) |

**Table S4.** Top 100 fungal taxa found in bioaerosol samples, based on and ordered by the mean relative abundance across bioaerosol samples. Relative abundances shown below for bioaerosol, foliar surface, and soil samples are the mean relative abundances for each indicated ASV within that sample type. Indicator type represents if the amplicon sequence variant (ASV) was an “indicator species” (*sensu* De Cáceres and Legendre 2009) for foliar surface samples, soil samples, or neither (labeled “not”). “Bioaerosol sample occupancy (%)” represents the percentage of samples, out of the 110 fungal bioaerosol samples retained following bioinformatics, which contained the ASV.

| **ASV #** | **Bio-aerosol rel. abund.**  **(%)** | **Indi-cator**  **Type** | **Bio-aerosol sample**  **occu-pancy**  **(%)** | **Soil sample**  **occu-pancy**  **(%)** | **Foliar sample**  **occu-pancy (%)** | **Phylum** | **Order** | **Family** | **Genus** | **Species** |
| --- | --- | --- | --- | --- | --- | --- | --- | --- | --- | --- |
| 2 | 5.75 | foliar | 100 | 0 | 89.8 | Basidiomycota | Agaricomycetes | NA | NA | NA |
| 3 | 4.77 | foliar | 100 | 1.3 | 88.1 | Basidiomycota | Agaricomycetes | Wrightoporiaceae | Wrightoporia | austrosinensis |
| 4 | 4.02 | foliar | 100 | 1.9 | 79.7 | Basidiomycota | Agaricomycetes | Polyporaceae | NA | NA |
| 11 | 1.94 | foliar | 100 | 0 | 42.4 | Basidiomycota | Agaricomycetes | Polyporaceae | Trametes | cubensis |
| 18 | 1.41 | foliar | 100 | 0 | 47.5 | Basidiomycota | Agaricomycetes | Polyporaceae | NA | NA |
| 19 | 1.28 | foliar | 100 | 0 | 71.2 | Basidiomycota | Agaricomycetes | Meruliaceae | NA | NA |
| 22 | 1.18 | foliar | 100 | 2.6 | 47.5 | Basidiomycota | Agaricomycetes | Polyporaceae | Trametes | cubensis |
| 26 | 1.17 | foliar | 100 | 0 | 49.2 | Basidiomycota | Agaricomycetes | Polyporaceae | Fomitella | supina |
| 28 | 1.15 | foliar | 99.1 | 0 | 59.3 | Basidiomycota | Agaricomycetes | Hymenochaetaceae | Phellinus | gilvus |
| 32 | 1.1 | foliar | 97.3 | 0 | 6.8 | Basidiomycota | Agaricomycetes | Fomitopsidaceae | Skeletocutis | chrysella |
| 16 | 1.08 | foliar | 76.4 | 1.9 | 91.5 | Basidiomycota | Agaricomycetes | Schizophyllaceae | Schizophyllum | commune |
| 35 | 0.96 | foliar | 95.5 | 0 | 33.9 | Basidiomycota | Agaricomycetes | Steccherinaceae | Ceriporiopsis | carnegieae |
| 37 | 0.9 | foliar | 96.4 | 0 | 69.5 | Basidiomycota | Agaricomycetes | Hymenochaetales_fam_Incertae_sedis | Trichaptum | sector |
| 29 | 0.87 | foliar | 79.1 | 0 | 88.1 | Basidiomycota | Agaricomycetes | Stereaceae | Stereum | NA |
| 43 | 0.87 | foliar | 99.1 | 0 | 81.4 | Basidiomycota | Agaricomycetes | Ganodermataceae | NA | NA |
| 27 | 0.76 | foliar | 50.9 | 1.9 | 59.3 | Basidiomycota | Agaricomycetes | Exidiaceae | Exidia | NA |
| 24 | 0.76 | foliar | 71.8 | 28.4 | 89.8 | Basidiomycota | Agaricomycetes | Ceratobasidiaceae | Ceratobasidium | NA |
| 14 | 0.75 | foliar | 100 | 54.2 | 100 | Ascomycota | Dothideomycetes | Cladosporiaceae | Cladosporium | delicatulum |
| 39 | 0.75 | foliar | 91.8 | 2.6 | 71.2 | Basidiomycota | Agaricomycetes | Hymenochaetales_fam_Incertae_sedis | Trichaptum | NA |
| 57 | 0.74 | foliar | 96.4 | 0.6 | 39 | Basidiomycota | Agaricomycetes | Meruliaceae | Phlebia | serialis |
| 56 | 0.74 | not | 94.5 | 0 | 0 | Basidiomycota | Agaricomycetes | Fomitopsidaceae | Melanoporia | nigra |
| 36 | 0.73 | foliar | 78.2 | 4.5 | 66.1 | Basidiomycota | Agaricomycetes | Cantharellales_fam_Incertae_sedis | Sistotrema | brinkmannii |
| 59 | 0.72 | foliar | 98.2 | 0 | 15.3 | Basidiomycota | Agaricomycetes | Fomitopsidaceae | Antrodia | oleracea |
| 25 | 0.7 | foliar | 80 | 1.3 | 98.3 | Basidiomycota | Agaricomycetes | Peniophoraceae | Peniophora | laxitexta |
| 49 | 0.66 | foliar | 79.1 | 0.6 | 78 | Basidiomycota | Agaricomycetes | Phanerochaetaceae | Phanerochaete | NA |
| 65 | 0.65 | foliar | 99.1 | 0 | 50.8 | Basidiomycota | Agaricomycetes | Steccherinaceae | Nigroporus | vinosus |
| 42 | 0.63 | foliar | 62.7 | 11 | 62.7 | Basidiomycota | Agaricomycetes | Ceratobasidiaceae | Ceratobasidium | NA |
| 48 | 0.62 | foliar | 72.7 | 0.6 | 71.2 | Basidiomycota | Agaricomycetes | NA | NA | NA |
| 47 | 0.57 | foliar | 64.5 | 0.6 | 81.4 | Basidiomycota | Agaricomycetes | Exidiaceae | Exidia | glandulosa |
| 74 | 0.57 | foliar | 97.3 | 0 | 47.5 | Basidiomycota | Agaricomycetes | Hymenochaetales_fam_Incertae_sedis | Resinicium | friabile |
| 78 | 0.54 | not | 96.4 | 0 | 1.7 | Basidiomycota | Agaricomycetes | Fomitopsidaceae | Skeletocutis | diluta |
| 60 | 0.51 | not | 83.6 | 1.9 | 45.8 | Basidiomycota | Agaricomycetes | Trechisporales_fam_Incertae_sedis | Sistotremastrum | niveocremeum |
| 86 | 0.5 | not | 89.1 | 0 | 0 | Basidiomycota | Agaricomycetes | Fomitopsidaceae | Melanoporia | nigra |
| 64 | 0.49 | foliar | 72.7 | 2.6 | 76.3 | Basidiomycota | Agaricomycetes | Hymenochaetales_fam_Incertae_sedis | Trichaptum | NA |
| 61 | 0.49 | foliar | 68.2 | 28.4 | 84.7 | Basidiomycota | Agaricomycetes | Ceratobasidiaceae | Ceratobasidium | NA |
| 85 | 0.47 | foliar | 85.5 | 0 | 54.2 | Basidiomycota | Agaricomycetes | Polyporaceae | Trametes | hirsuta |
| 100 | 0.46 | foliar | 97.3 | 0 | 49.2 | Basidiomycota | Agaricomycetes | Hymenochaetales_fam_Incertae_sedis | Skvortzovia | furfuracea |
| 90 | 0.45 | foliar | 96.4 | 0 | 50.8 | Basidiomycota | Agaricomycetes | Meruliaceae | NA | NA |
| 105 | 0.44 | foliar | 90 | 0 | 52.5 | Basidiomycota | Agaricomycetes | Meruliaceae | Scopuloides | NA |
| 71 | 0.43 | foliar | 82.7 | 0 | 78 | Basidiomycota | Agaricomycetes | Phanerochaetaceae | Phlebiopsis | castanea |
| 88 | 0.41 | foliar | 97.3 | 0.6 | 67.8 | Basidiomycota | Agaricomycetes | Ganodermataceae | Perenniporia | truncatospora |
| 45 | 0.41 | foliar | 90.9 | 51.6 | 100 | Ascomycota | Dothideomycetes | Pleosporaceae | Alternaria | angustiovoidea |
| 82 | 0.39 | foliar | 79.1 | 0 | 88.1 | Basidiomycota | Agaricomycetes | Phanerochaetaceae | Phlebiopsis | NA |
| 121 | 0.39 | foliar | 94.5 | 1.3 | 44.1 | Basidiomycota | Agaricomycetes | Polyporaceae | Fomes | fasciatus |
| 83 | 0.38 | foliar | 61.8 | 0 | 69.5 | Basidiomycota | Agaricomycetes | NA | NA | NA |
| 109 | 0.38 | foliar | 90 | 0.6 | 50.8 | Basidiomycota | Agaricomycetes | Phanerochaetaceae | Phanerochaete | NA |
| 152 | 0.38 | foliar | 95.5 | 0 | 44.1 | Basidiomycota | Agaricomycetes | Meruliaceae | Phlebia | rufa |
| 130 | 0.38 | foliar | 86.4 | 0 | 6.8 | Basidiomycota | Agaricomycetes | Fomitopsidaceae | Melanoporia | nigra |
| 142 | 0.38 | foliar | 96.4 | 0 | 27.1 | Basidiomycota | Agaricomycetes | Hymenochaetaceae | Phellinus | gilvus |
| 115 | 0.37 | foliar | 90.9 | 0 | 50.8 | Basidiomycota | Agaricomycetes | Hymenochaetales_fam_Incertae_sedis | Trichaptum | sector |
| 124 | 0.37 | foliar | 96.4 | 0 | 25.4 | Basidiomycota | Agaricomycetes | Hymenochaetaceae | Phellinus | gilvus |
| 87 | 0.37 | foliar | 78.2 | 0 | 84.7 | Basidiomycota | Agaricomycetes | Phanerochaetaceae | Phlebiopsis | NA |
| 131 | 0.36 | not | 99.1 | 0 | 0 | Basidiomycota | Agaricomycetes | Polyporaceae | Lentinus | crinitus |
| 108 | 0.36 | foliar | 75.5 | 0 | 88.1 | Basidiomycota | Agaricomycetes | Irpicaceae | NA | NA |
| 139 | 0.34 | foliar | 94.5 | 0 | 32.2 | Basidiomycota | Agaricomycetes | Meruliaceae | Phlebia | NA |
| 137 | 0.33 | foliar | 94.5 | 0 | 55.9 | Basidiomycota | Agaricomycetes | Schizoporaceae | Hyphodontia | NA |
| 173 | 0.33 | foliar | 88.2 | 0 | 23.7 | Basidiomycota | Agaricomycetes | NA | NA | NA |
| 154 | 0.33 | foliar | 96.4 | 1.3 | 50.8 | Basidiomycota | Agaricomycetes | Auriscalpiaceae | Gloeodontia | eriobotryae |
| 169 | 0.32 | not | 91.8 | 0 | 3.4 | Basidiomycota | Agaricomycetes | Fomitopsidaceae | Skeletocutis | odora |
| 84 | 0.31 | foliar | 63.6 | 11.6 | 86.4 | Basidiomycota | Agaricomycetes | Peniophoraceae | Peniophora | NA |
| 110 | 0.31 | foliar | 72.7 | 0 | 76.3 | Basidiomycota | Agaricomycetes | Stereaceae | Stereum | hirsutum |
| 159 | 0.3 | foliar | 78.2 | 0 | 28.8 | Basidiomycota | Agaricomycetes | Fomitopsidaceae | Antrodia | ladiana |
| 155 | 0.3 | foliar | 87.3 | 0 | 54.2 | Basidiomycota | Agaricomycetes | Meruliaceae | Phlebia | NA |
| 101 | 0.3 | foliar | 67.3 | 1.9 | 83.1 | Basidiomycota | Agaricomycetes | Stereaceae | Stereum | complicatum |
| 133 | 0.3 | foliar | 68.2 | 0 | 49.2 | Basidiomycota | Agaricomycetes | Exidiaceae | Eichleriella | bactriana |
| 162 | 0.29 | foliar | 84.5 | 0 | 27.1 | Basidiomycota | Agaricomycetes | Hymenochaetales_fam_Incertae_sedis | Resinicium | rimulosum |
| 117 | 0.29 | foliar | 74.5 | 0 | 83.1 | Basidiomycota | Agaricomycetes | Stereaceae | Stereum | NA |
| 145 | 0.29 | foliar | 83.6 | 0 | 62.7 | Basidiomycota | Agaricomycetes | Hyphodermataceae | Hyphoderma | roseocremeum |
| 167 | 0.28 | foliar | 91.8 | 0 | 44.1 | Basidiomycota | Agaricomycetes | NA | NA | NA |
| 129 | 0.28 | foliar | 70.9 | 0 | 55.9 | Basidiomycota | Agaricomycetes | NA | NA | NA |
| 96 | 0.26 | foliar | 61.8 | 5.2 | 91.5 | Basidiomycota | Agaricomycetes | Peniophoraceae | Peniophora | NA |
| 111 | 0.26 | foliar | 57.3 | 6.5 | 72.9 | Basidiomycota | Agaricomycetes | Phanerochaetaceae | Hyphodermella | NA |
| 198 | 0.25 | foliar | 80 | 0 | 15.3 | Basidiomycota | Agaricomycetes | Cantharellales_fam_Incertae_sedis | Burgoa | verzuoliana |
| 200 | 0.25 | foliar | 92.7 | 0 | 23.7 | Basidiomycota | Agaricomycetes | Steccherinaceae | NA | NA |
| 181 | 0.25 | foliar | 80 | 0 | 57.6 | Basidiomycota | Agaricomycetes | Phanerochaetaceae | Hyphodermella | NA |
| 112 | 0.24 | foliar | 60.9 | 0 | 86.4 | Basidiomycota | Agaricomycetes | Peniophoraceae | Peniophora | NA |
| 201 | 0.24 | foliar | 90 | 0 | 32.2 | Basidiomycota | Agaricomycetes | Hymenochaetales_fam_Incertae_sedis | Resinicium | friabile |
| 225 | 0.24 | foliar | 89.1 | 1.3 | 44.1 | Basidiomycota | Agaricomycetes | NA | NA | NA |
| 119 | 0.23 | foliar | 67.3 | 0 | 88.1 | Basidiomycota | Agaricomycetes | Peniophoraceae | Peniophora | laxitexta |
| 233 | 0.23 | foliar | 91.8 | 0 | 40.7 | Basidiomycota | Agaricomycetes | NA | NA | NA |
| 179 | 0.22 | not | 71.8 | 2.6 | 6.8 | Basidiomycota | Agaricomycetes | Trechisporales_fam_Incertae_sedis | Sistotremastrum | suecicum |
| 231 | 0.22 | foliar | 55.5 | 0 | 32.2 | Basidiomycota | Agaricomycetes | Pleurotaceae | Pleurotus | pulmonarius |
| 217 | 0.22 | not | 84.5 | 6.5 | 25.4 | Basidiomycota | Agaricomycetes | Fomitopsidaceae | Cinereomyces | lindbladii |
| 219 | 0.22 | foliar | 81.8 | 0 | 13.6 | Basidiomycota | Agaricomycetes | Hymenochaetales_fam_Incertae_sedis | Resinicium | rimulosum |
| 232 | 0.22 | foliar | 86.4 | 0 | 35.6 | Basidiomycota | Agaricomycetes | Meruliaceae | Mycoacia | fuscoatra |
| 180 | 0.22 | foliar | 66.4 | 0 | 44.1 | Basidiomycota | Agaricomycetes | Schizoporaceae | NA | NA |
| 193 | 0.21 | foliar | 56.4 | 1.9 | 28.8 | Basidiomycota | NA | NA | NA | NA |
| 245 | 0.21 | foliar | 88.2 | 0 | 6.8 | Basidiomycota | Agaricomycetes | Trechisporales_fam_Incertae_sedis | Sistotremastrum | guttuliferum |
| 190 | 0.21 | foliar | 70 | 0 | 72.9 | Basidiomycota | Agaricomycetes | Stereaceae | Stereum | NA |
| 189 | 0.21 | foliar | 74.5 | 0 | 69.5 | Basidiomycota | Agaricomycetes | Irpicaceae | Irpex | hydnoides |
| 275 | 0.2 | not | 70.9 | 0 | 0 | Basidiomycota | Agaricomycetes | Fomitopsidaceae | Melanoporia | nigra |
| 221 | 0.2 | foliar | 66.4 | 0 | 59.3 | Basidiomycota | Agaricomycetes | Exidiaceae | Eichleriella | bactriana |
| 203 | 0.2 | foliar | 70.9 | 0.6 | 47.5 | Basidiomycota | Agaricomycetes | Steccherinaceae | Steccherinum | NA |
| 274 | 0.19 | foliar | 76.4 | 0 | 5.1 | Basidiomycota | Agaricomycetes | NA | NA | NA |
| 141 | 0.18 | foliar | 78.2 | 21.3 | 96.6 | Ascomycota | Dothideomycetes | Lophiostomataceae | NA | NA |
| 204 | 0.18 | foliar | 60.9 | 1.9 | 88.1 | Basidiomycota | Microbotryomycetes | Sporidiobolaceae | Sporobolomyces | patagonicus |
| 185 | 0.18 | foliar | 46.4 | 0 | 67.8 | Basidiomycota | Agaricomycetes | Tricholomataceae | Resupinatus | americanus |
| 281 | 0.17 | foliar | 92.7 | 0 | 15.3 | Basidiomycota | Agaricomycetes | Phanerochaetaceae | Phanerochaete | NA |
| 300 | 0.17 | foliar | 87.3 | 0 | 42.4 | Basidiomycota | Agaricomycetes | Meruliaceae | NA | NA |
| 205 | 0.17 | foliar | 59.1 | 0 | 55.9 | Basidiomycota | Agaricomycetes | Schizophyllaceae | Schizophyllum | commune |

**Table S5.** Top 100 bacterial taxa (as amplicon sequence variants, ASVs) found in bioaerosol samples. Bioaerosol relative abundance values shown are the mean relative abundances for each indicated ASV across bioaerosol samples, and ASVs are ordered based on these mean relative abundances. Indicator type represents if the amplicon sequence variant (ASV) was an “indicator species” (*sensu* De Cáceres and Legendre 2009) for foliar surface samples, soil samples, or neither (labeled “not”). Percentage sample occupancies for each type of sample indicate the percentage of bioaerosol (n=84 samples), foliar surface (n=58), or soil samples (n=157) in which an ASV was detected.

| **ASV #** | **Bio-aerosol rel. abund.**  **(%)** | **Indi-cator**  **Type** | **Bio-aerosol sample**  **occu-pancy**  **(%)** | **Soil sample**  **occu-pancy**  **(%)** | **Foliar sample**  **occu-pancy (%)** | **Phylum** | **Class** | **Family** | **Genus** |
| --- | --- | --- | --- | --- | --- | --- | --- | --- | --- |
| 10 | 2.1 | not | 72.6 | 0 | 0 | Firmicutes | Bacilli | Staphylococcaceae | Staphylococcus |
| 49 | 1.25 | foliar | 13.1 | 0 | 15.5 | Myxococcota | Myxococcia | Myxococcaceae | Cystobacter |
| 86 | 1.11 | not | 59.5 | 0 | 1.7 | Proteobacteria | Gammaproteobacteria | Burkholderiaceae | NA |
| 78 | 1.01 | foliar | 23.8 | 0 | 5.2 | Proteobacteria | Alphaproteobacteria | Sphingomonadaceae | Sphingobium |
| 11 | 0.92 | soil | 73.8 | 35 | 0 | Proteobacteria | Alphaproteobacteria | Beijerinckiaceae | Methylobacterium-Methylorubrum |
| 173 | 0.86 | foliar | 13.1 | 0 | 6.9 | Proteobacteria | NA | NA | NA |
| 126 | 0.84 | not | 32.1 | 0 | 0 | Deinococcota | Deinococci | Deinococcaceae | Deinococcus |
| 4 | 0.77 | foliar | 57.1 | 4.5 | 100 | Proteobacteria | Alphaproteobacteria | Beijerinckiaceae | Methylobacterium-Methylorubrum |
| 48 | 0.7 | not | 19 | 0 | 0 | Myxococcota | Myxococcia | Myxococcaceae | Cystobacter |
| 69 | 0.66 | foliar | 29.8 | 1.3 | 13.8 | Proteobacteria | Gammaproteobacteria | Moraxellaceae | Acinetobacter |
| 206 | 0.63 | foliar | 17.9 | 0 | 5.2 | Proteobacteria | Alphaproteobacteria | Rhodobacteraceae | Paracoccus |
| 177 | 0.59 | foliar | 16.7 | 17.8 | 34.5 | Proteobacteria | Gammaproteobacteria | Oxalobacteraceae | Massilia |
| 192 | 0.56 | foliar | 17.9 | 0 | 79.3 | Planctomycetota | Planctomycetes | Isosphaeraceae | Tundrisphaera |
| 201 | 0.53 | not | 42.9 | 11.5 | 25.9 | Firmicutes | Bacilli | Bacillaceae | Bacillus |
| 65 | 0.52 | soil | 29.8 | 99.4 | 56.9 | Proteobacteria | Alphaproteobacteria | Xanthobacteraceae | Bradyrhizobium |
| 129 | 0.52 | not | 29.8 | 0 | 0 | Firmicutes | Bacilli | Lactobacillaceae | Ligilactobacillus |
| 270 | 0.5 | foliar | 19 | 0 | 24.1 | Proteobacteria | Alphaproteobacteria | AB1 | NA |
| 63 | 0.48 | foliar | 52.4 | 1.9 | 93.1 | Actinobacteriota | Actinobacteria | Microbacteriaceae | Curtobacterium |
| 142 | 0.47 | not | 33.3 | 0 | 0 | Firmicutes | Clostridia | Peptostreptococcaceae | Romboutsia |
| 146 | 0.46 | not | 36.9 | 0 | 0 | Firmicutes | Bacilli | Staphylococcaceae | Salinicoccus |
| 132 | 0.45 | not | 48.8 | 0 | 0 | Firmicutes | Bacilli | Staphylococcaceae | Staphylococcus |
| 171 | 0.43 | foliar | 38.1 | 0 | 5.2 | Actinobacteriota | Actinobacteria | Brevibacteriaceae | Brevibacterium |
| 231 | 0.42 | not | 35.7 | 0 | 0 | Firmicutes | Bacilli | Bacillaceae | NA |
| 663 | 0.41 | not | 2.4 | 0 | 0 | Proteobacteria | Alphaproteobacteria | Rickettsiaceae | NA |
| 117 | 0.41 | foliar | 36.9 | 0.6 | 82.8 | Actinobacteriota | Actinobacteria | Microbacteriaceae | Curtobacterium |
| 237 | 0.35 | not | 22.6 | 74.5 | 41.4 | Firmicutes | Bacilli | Bacillaceae | Bacillus |
| 193 | 0.35 | foliar | 42.9 | 0.6 | 55.2 | Firmicutes | Bacilli | Staphylococcaceae | Staphylococcus |
| 239 | 0.33 | not | 48.8 | 0.6 | 0 | Firmicutes | Bacilli | Bacillaceae | Anoxybacillus |
| 33 | 0.32 | foliar | 44 | 0 | 98.3 | Proteobacteria | Alphaproteobacteria | Sphingomonadaceae | Sphingomonas |
| 1083 | 0.31 | not | 1.2 | 0 | 3.4 | Proteobacteria | NA | NA | NA |
| 856 | 0.31 | foliar | 8.3 | 0 | 36.2 | Planctomycetota | Planctomycetes | Isosphaeraceae | Tundrisphaera |
| 199 | 0.3 | not | 44 | 0 | 1.7 | Firmicutes | Bacilli | Lactobacillaceae | Lactobacillus |
| 678 | 0.29 | foliar | 7.1 | 0 | 5.2 | Proteobacteria | Gammaproteobacteria | Neisseriaceae | NA |
| 160 | 0.29 | not | 29.8 | 5.7 | 3.4 | Deinococcota | Deinococci | Thermaceae | Meiothermus |
| 104 | 0.29 | foliar | 28.6 | 0 | 31 | Proteobacteria | Gammaproteobacteria | NA | NA |
| 184 | 0.28 | foliar | 1.2 | 0 | 10.3 | Myxococcota | Myxococcia | Myxococcaceae | Melittangium |
| 359 | 0.28 | not | 22.6 | 0 | 0 | Firmicutes | Bacilli | Staphylococcaceae | NA |
| 469 | 0.28 | foliar | 6 | 0 | 67.2 | Planctomycetota | Planctomycetes | Isosphaeraceae | Tundrisphaera |
| 198 | 0.28 | not | 32.1 | 0 | 0 | Firmicutes | Bacilli | Bacillaceae | Pseudogracilibacillus |
| 1576 | 0.28 | not | 1.2 | 0 | 0 | Proteobacteria | Alphaproteobacteria | Rickettsiaceae | NA |
| 188 | 0.27 | foliar | 34.5 | 20.4 | 82.8 | Proteobacteria | Gammaproteobacteria | Oxalobacteraceae | Massilia |
| 986 | 0.27 | not | 2.4 | 0 | 0 | Proteobacteria | Gammaproteobacteria | Neisseriaceae | NA |
| 1149 | 0.27 | not | 3.6 | 0 | 0 | Proteobacteria | Gammaproteobacteria | Piscirickettsiaceae | Candidatus Endoecteinascidia |
| 468 | 0.27 | not | 13.1 | 0 | 1.7 | Proteobacteria | Gammaproteobacteria | Moraxellaceae | Acinetobacter |
| 90 | 0.26 | not | 19 | 3.8 | 5.2 | Deinococcota | Deinococci | Deinococcaceae | Deinococcus |
| 961 | 0.26 | not | 20.2 | 1.9 | 0 | Deinococcota | Deinococci | Deinococcaceae | Deinococcus |
| 1393 | 0.26 | foliar | 1.2 | 0 | 12.1 | Proteobacteria | Alphaproteobacteria | Caedibacteraceae | Caedibacter |
| 320 | 0.26 | foliar | 17.9 | 0 | 13.8 | Proteobacteria | Alphaproteobacteria | Rhizobiaceae | Neorhizobium |
| 781 | 0.26 | not | 2.4 | 0 | 0 | Firmicutes | Bacilli | Thermicanaceae | Hydrogenibacillus |
| 148 | 0.26 | foliar | 20.2 | 0 | 89.7 | Proteobacteria | Alphaproteobacteria | Beijerinckiaceae | 1174-901-12 |
| 1187 | 0.26 | not | 1.2 | 0 | 0 | Proteobacteria | Alphaproteobacteria | Rickettsiaceae | NA |
| 533 | 0.25 | not | 7.1 | 20.4 | 17.2 | Firmicutes | Bacilli | Planococcaceae | NA |
| 261 | 0.25 | foliar | 25 | 0 | 5.2 | Firmicutes | Bacilli | Aerococcaceae | Aerococcus |
| 311 | 0.25 | foliar | 14.3 | 0 | 6.9 | Proteobacteria | Gammaproteobacteria | Moraxellaceae | Enhydrobacter |
| 406 | 0.25 | foliar | 10.7 | 0 | 6.9 | Proteobacteria | Gammaproteobacteria | Moraxellaceae | Acinetobacter |
| 84 | 0.25 | foliar | 27.4 | 0 | 100 | Proteobacteria | Alphaproteobacteria | Beijerinckiaceae | 1174-901-12 |
| 280 | 0.25 | foliar | 19 | 0 | 27.6 | Proteobacteria | Alphaproteobacteria | Sphingomonadaceae | Sphingomonas |
| 70 | 0.24 | foliar | 32.1 | 33.8 | 94.8 | Proteobacteria | Gammaproteobacteria | Oxalobacteraceae | Massilia |
| 407 | 0.24 | not | 26.2 | 28.7 | 24.1 | Actinobacteriota | Actinobacteria | Streptomycetaceae | Streptomyces |
| 298 | 0.24 | not | 19 | 0 | 1.7 | Actinobacteriota | Actinobacteria | Micrococcaceae | Micrococcus |
| 315 | 0.24 | foliar | 39.3 | 2.5 | 24.1 | Firmicutes | Bacilli | NA | NA |
| 877 | 0.24 | foliar | 6 | 0 | 10.3 | Actinobacteriota | Actinobacteria | Corynebacteriaceae | Lawsonella |
| 654 | 0.23 | foliar | 10.7 | 0 | 20.7 | Planctomycetota | Planctomycetes | Isosphaeraceae | Tundrisphaera |
| 262 | 0.23 | foliar | 23.8 | 11.5 | 51.7 | Proteobacteria | Gammaproteobacteria | Oxalobacteraceae | Massilia |
| 344 | 0.23 | foliar | 8.3 | 25.5 | 43.1 | Actinobacteriota | Actinobacteria | Micromonosporaceae | Actinoplanes |
| 310 | 0.23 | foliar | 22.6 | 4.5 | 70.7 | Actinobacteriota | Actinobacteria | Microbacteriaceae | Frondihabitans |
| 966 | 0.23 | not | 3.6 | 0.6 | 0 | Proteobacteria | Alphaproteobacteria | Paracaedibacteraceae | Candidatus Captivus |
| 174 | 0.23 | foliar | 19 | 8.9 | 89.7 | Acidobacteriota | Acidobacteriae | Acidobacteriaceae (Subgroup 1) | Bryocella |
| 591 | 0.23 | foliar | 10.7 | 0 | 13.8 | Proteobacteria | Alphaproteobacteria | Sphingomonadaceae | Novosphingobium |
| 1120 | 0.23 | not | 2.4 | 0 | 0 | Proteobacteria | Alphaproteobacteria | Sphingomonadaceae | Altererythrobacter |
| 513 | 0.22 | not | 9.5 | 0 | 3.4 | Proteobacteria | Alphaproteobacteria | Paracaedibacteraceae | Candidatus Paracaedibacter |
| 355 | 0.22 | soil | 3.6 | 73.2 | 27.6 | Bacteroidota | Bacteroidia | Chitinophagaceae | Flavisolibacter |
| 20 | 0.22 | foliar | 42.9 | 6.4 | 100 | Proteobacteria | Alphaproteobacteria | Beijerinckiaceae | 1174-901-12 |
| 289 | 0.22 | not | 23.8 | 0 | 0 | Firmicutes | Bacilli | Bacillaceae | NA |
| 380 | 0.22 | foliar | 17.9 | 0 | 29.3 | Proteobacteria | Alphaproteobacteria | Sphingomonadaceae | Sphingomonas |
| 202 | 0.22 | foliar | 23.8 | 0 | 79.3 | Proteobacteria | Alphaproteobacteria | Acetobacteraceae | Acidisoma |
| 253 | 0.22 | foliar | 9.5 | 0 | 17.2 | Proteobacteria | NA | NA | NA |
| 1976 | 0.22 | not | 2.4 | 0 | 0 | Bacteroidota | Bacteroidia | Prevotellaceae | Prevotella_9 |
| 361 | 0.22 | not | 27.4 | 0 | 0 | Actinobacteriota | Actinobacteria | Dermabacteraceae | Brachybacterium |
| 1565 | 0.22 | not | 1.2 | 0 | 0 | Firmicutes | Clostridia | Lachnospiraceae | [Ruminococcus] torques group |
| 658 | 0.21 | foliar | 10.7 | 0 | 6.9 | Firmicutes | Bacilli | Streptococcaceae | Streptococcus |
| 865 | 0.21 | foliar | 2.4 | 0 | 13.8 | Proteobacteria | Gammaproteobacteria | Pasteurellaceae | Haemophilus |
| 930 | 0.21 | not | 14.3 | 0 | 0 | Firmicutes | Bacilli | Bacillaceae | NA |
| 395 | 0.21 | not | 9.5 | 0 | 0 | Proteobacteria | Gammaproteobacteria | Pseudomonadaceae | Pseudomonas |
| 810 | 0.21 | not | 4.8 | 0 | 0 | Proteobacteria | Alphaproteobacteria | AB1 | NA |
| 1201 | 0.21 | not | 7.1 | 0 | 0 | Proteobacteria | Alphaproteobacteria | Sphingomonadaceae | Sphingobium |
| 1221 | 0.21 | foliar | 3.6 | 0 | 20.7 | Proteobacteria | Alphaproteobacteria | Rickettsiaceae | Candidatus Trichorickettsia |
| 944 | 0.21 | foliar | 2.4 | 0 | 8.6 | Cyanobacteria | Cyanobacteriia | Nostocaceae | Scytonema VB-61278 |
| 116 | 0.2 | foliar | 23.8 | 30.6 | 93.1 | Proteobacteria | Alphaproteobacteria | Sphingomonadaceae | Sphingomonas |
| 222 | 0.2 | foliar | 20.2 | 12.1 | 91.4 | Actinobacteriota | Actinobacteria | Microbacteriaceae | Amnibacterium |
| 627 | 0.2 | foliar | 15.5 | 0 | 8.6 | Proteobacteria | Gammaproteobacteria | Pseudomonadaceae | Pseudomonas |
| 1162 | 0.2 | not | 2.4 | 0 | 0 | Verrucomicrobiota | Verrucomicrobiae | Chthoniobacteraceae | Chthoniobacter |
| 699 | 0.2 | not | 3.6 | 0 | 0 | Actinobacteriota | Actinobacteria | Micrococcaceae | Kocuria |
| 820 | 0.2 | foliar | 6 | 10.2 | 22.4 | Proteobacteria | Alphaproteobacteria | Acetobacteraceae | Acidocella |
| 101 | 0.2 | foliar | 17.9 | 0 | 98.3 | Proteobacteria | Alphaproteobacteria | Acetobacteraceae | Rhodopila |
| 660 | 0.2 | not | 14.3 | 0 | 0 | Firmicutes | Bacilli | Lactobacillaceae | Lactobacillus |
| 1068 | 0.19 | not | 7.1 | 0 | 0 | Proteobacteria | NA | NA | NA |
| 329 | 0.19 | soil | 22.6 | 7.6 | 0 | Proteobacteria | Gammaproteobacteria | Burkholderiaceae | Cupriavidus |
| 421 | 0.19 | not | 17.9 | 0 | 0 | Firmicutes | Bacilli | Staphylococcaceae | Jeotgalicoccus |
| 1754 | 0.19 | not | 3.6 | 0 | 0 | Firmicutes | Clostridia | Peptostreptococcaceae | Romboutsia |

**Table S6.** Results of PERMANOVAs (adonis2 function from vegan R package) performed to test the effect of habitat type (open patch or forested matrix) on Bray-Curtis dissimilarities among bioaerosol samples for fungal taxa (a) and bacterial taxa (b). To account for the nested design of habitat type within each experimental unit (EU), permutations were performed within each EU, and the potential significance of EU was tested before assessing the effect of habitat type. See main text for additional information on statistical design.

(a)

| **FUNGI (number of bioaerosol samples = 96)** | | | | | |
| --- | --- | --- | --- | --- | --- |
| **Variable** | **Degrees of freedom** | **Sum of Squares** | **R^2^** | **Pseudo-F** | **Pr(>F)** |
| **EU** | 3 | 3.87 | 0.19 | 6.96 | .810 |
| **Habitat type (open patch or forested matrix)** | 1 | 0.11 | 0.01 | 0.59 | .810 |
| **Residual** | 91 | 16.88 | .81 |  |  |

(b)

| **BACTERIA (number of bioaerosol samples = 56)** | | | | | |
| --- | --- | --- | --- | --- | --- |
| **Variable** | **Degrees of freedom** | **Sum of Squares** | **R^2^** | **Pseudo-F** | **Pr(>F)** |
| **EU** | 3 | 1.71 | .07 | 1.22 | .571 |
| **Habitat type (open patch or forested matrix)** | 1 | 0.45 | .02 | 0.98 | .571 |
| **Residual** | 51 | 23.71 | .92 |  |  |

**Table S7.** Spore volume matches by fungal order for full and subsetted spore volume datasets. Volumes were inferred by matching taxa at the species, or genus level if species not available, to entries in the database from Aguilar-Trigueros et al. (2023) and then taking the median of these matches. The subsetted spore volume dataset retains only ASVs for which there is minimal variation among volumes reported for each ASV; see main text methods for further information. Numbers below “Bioaerosols” and “Foliar surfaces” refer to the number of ASVs in a given order that were bioaerosol- or foliar surface-associated and for which spore volume information was available in the Aguilar-Trigueros et al. database.

|  | Full spore volume dataset | | | Subsetted spore volume dataset | | |
| --- | --- | --- | --- | --- | --- | --- |
| Fungal Order | Bioaerosols | Foliar surfaces | Median spore volume (μm) | Bioaerosols | Foliar surfaces | Median spore volume (μm) |
| Agaricales | 4 | 0 | 76.5 | 2 | 0 | 96.8 |
| Auriculariales | 1 | 0 | 17.4 | 1 | 0 | 17.4 |
| Cantharellales | 2 | 0 | 13.1 | NA | NA | NA |
| Capnodiales | 0 | 17 | 156.0 | 0 | 1 | 584.5 |
| Chaetothyriales | 0 | 10 | 1104.5 | 0 | 2 | 475.6 |
| Corticiales | 1 | 0 | 46.5 | NA | NA | NA |
| Dothideales | 0 | 1 | 198.5 | NA | NA | NA |
| Filobasidiales | 0 | 2 | 83.4 | NA | NA | NA |
| Gloeophyllales | 3 | 0 | 65.7 | NA | NA | NA |
| Hymenochaetales | 27 | 0 | 16.2 | 5 | 0 | 21.1 |
| Pleosporales | 0 | 19 | 254.0 | 0 | 7 | 254.0 |
| Polyporales | 61 | 0 | 25.7 | 12 | 0 | 29.4 |
| Russulales | 11 | 0 | 25.9 | 4 | 0 | 17.6 |
| Taphrinales | 0 | 11 | 38.9 | NA | NA | NA |
| Trechisporales | 6 | 0 | 17.4 | 1 | 0 | 48.1 |
| Tremellales | 0 | 14 | 30.5 | 0 | 10 | 30.5 |
| Venturiales | 0 | 1 | 42.4 | 0 | 1 | 42.4 |
| Xylariales | 0 | 1 | 606.1 | NA | NA | NA |

**Figure S1.** Plots showing (a) temperature, (b) wind, (c) humidity, and (d) total rainfall for each of the 16 sampling days, summarized over each 24-hour sampling period beginning on the date shown above. Each sampling day consisted of collecting bioaerosol samples in one of four experimental units (EU), given in Table S1. See Table S1 caption or main text for more information on weather data collection.

**Figure S2.** Non-metric multidimensional scaling (NMDS) ordinations based on Bray-Curtis dissimilarities among bioaerosol, foliar surface, and soil samples for fungal (a) and bacterial (b) datasets. Both fungal and bacterial communities are distinct among sample types (i.e., bioaerosol, foliar surface, and soil) based on PERMANOVAs with Bonferroni post-hoc corrections (*p* < .001 for all comparisons for both prokaryotes and fungi).

**Figure S3.** Proportions of taxa in bioaerosols and the potential source environments of foliar surfaces and soil. Panels (a) and (b) respectively show the proportion of taxa (based on presence or absence) from foliar surface or soil indicator taxa in each fungal and bacterial bioaerosol sample. Bioaerosol samples featured a larger proportion of distinct taxa from foliar surface than soil indicator taxa (Wilcoxon signed-rank tests: V = 6,105 and for fungi and V = 3,570 for bacteria, *p* < .001 for both).

**Figure S4.** Comparison of bioaerosols collected in the open patch and forested matrix in terms of ITS and I6S rRNA gene copies (a, c) and community composition (b, d). In panels (a) and (c), the number of gene copies detected in forested matrix and open patch samples did not differ for either ITS rRNA copies (i.e., fungi, χ^2^(1, N = 110) = 1.48, *p* = .22) or 16S rRNA copies (i.e., bacteria and archaea, χ^2^(1, N = 84) = 1.39, *p* = .24); see Methods in main text for more information on statistical tests. The non-metric multidimensional scaling (NMDS) ordinations shown in panels (b) and (d) are based on Bray-Curtis dissimilarities among bioaerosol samples. Bioaerosol samples did not differ with respect to habitat type (PERMANOVAs: *p* = .81 for fungi and *p* = .57 for bacteria, see text and Table S6 for more details).
